# Therapeutic activity of a hematopoietic stem cell-delivered cell-penetrating frataxin in Friedreich’s ataxia models

**DOI:** 10.1016/j.xcrm.2026.102803

**Published:** 2026-05-13

**Authors:** Jeffrey Pido-Lopez, Shefta E Moula, Enas Shaban, Konstantinos Stamatiou, Bethan J. Critchley, Thomas E. Whittaker, Stina Svensson, Sara Anjomani-Virmouni, Ester Kalef-Ezra, Lucinda Carr, Jane Hassel, Adrian J. Thrasher, Manju A. Kurian, Ian A. Blair, Teerapat Rojsajjakul, Giorgia Santilli, Arturo Sala

**Affiliations:** 1 Centre for Inflammation Research and Translational Medicine, Brunel, University of London, London UB8 3PH, UK; 2 Infection, Immunity and Inflammation Research and Teaching Department, University College London, Great Ormond Street Institute of Child Health, London, UK; 3 Centre for Genome Engineering and Maintenance, College of Health, Medicine and Life Sciences, Brunel, University of London, London, UK; 4 Molecular Neurosciences, Developmental Neurosciences Programme, UCL Institute of Child Health, London, UK; 5 Department of Neurology, Great Ormond Street Hospital for Children NHS Foundation Trust, London, UK; 6 Department of Systems Pharmacology and Translational Therapeutics, Perelman School of Medicine, University of Pennsylvania, Philadelphia, PA 19104, USA

**Keywords:** frataxin, cell and gene therapy, ataxia, mitochondria, behavioural assays, cell-penetrating peptide, transplantation, hematopoietic stem cells, secretory peptide.

## Abstract

Friedreich’s ataxia (FRDA) is an autosomal recessive neurodegenerative disease caused by a GAA repeat expansion in the *frataxin* (*FXN*) gene, leading to reduced frataxin, a protein essential for mitochondrial function. We developed a replacement strategy using a fusion protein containing secretion and cell-penetrating sequences fused to the frataxin precursor. *In vitro* studies confirmed secretion, cellular penetration, mitochondrial localization, and rescue of biochemical defects and apoptosis in cells from patients with FRDA. The therapeutic cDNA was cloned into a lentiviral vector and used to transduce hematopoietic stem and progenitor cells (HSPCs) from YG8sR mice, an FRDA model. Autologous transplantation of modified HSPCs produced stable peptide secretion in the bloodstream and delayed the onset of motor coordination symptoms, accompanied by improved biochemical and anatomical parameters. Patient-derived CD34^+^ HSPCs transduced with the vector differentiated normally into macrophages and secreted the peptide. These results support a cell and gene therapy strategy for long-term stabilization of FRDA.

## Highlights

• Reduced levels of the mitochondrial protein frataxin cause FRDA
• HSPCs have been engineered to secrete a cell-penetrating frataxin protein
• Autologous transplantation of modified HSPCs ameliorates symptoms in FRDA mice
• Patient HSPCs modified by gene therapy secrete the therapeutic frataxin protein

## Graphical abstract

**Figure:**
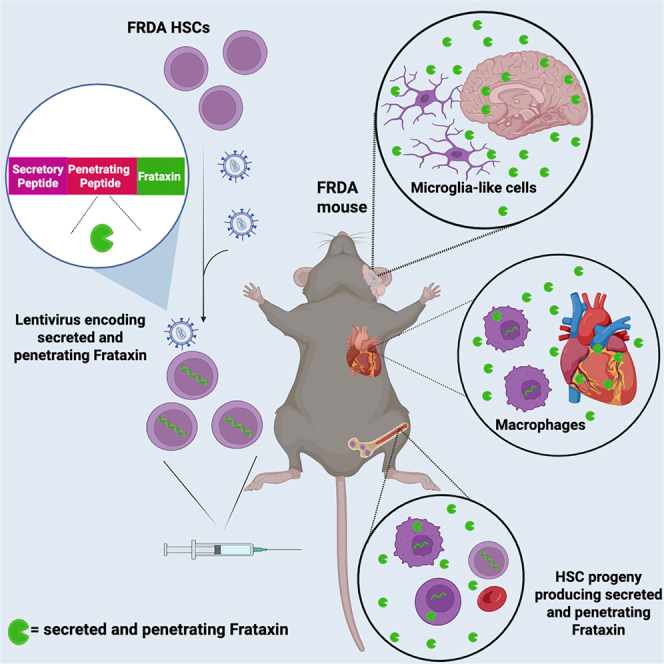

## Teaser

Engineered hematopoietic stem cells delivering a secreted, tissue-penetrating frataxin restore mitochondrial function and ameliorate disease features in Friedreich’s ataxia models. This gene and cell therapy approach enables sustained systemic protein delivery, improving motor performance and tissue pathology, and supports a translational strategy for long-term disease stabilization.

## Introduction

Friedreich’s ataxia (FRDA) is an autosomal recessive disease characterized by progressive spinocerebellar neuropathy, ataxia, muscle atrophy, diabetes, and cardiomyopathy.^1^ Affected individuals show severely reduced cellular levels of the iron-binding mitochondrial protein frataxin (FXN), which is brought about by the hyperexpansion of a GAA trinucleotide repeat sequence within the first intron of the FXN gene (*FXN*) on chromosome 9, leading to transcriptional repression of *FXN*^2^^,^^3^ *.* Deficiency of FXN results in elevated oxidative stress and accumulation of iron within mitochondria, leading to mitochondrial disfunction.^4^^,^^5^ Currently, an efficacious treatment for FRDA is absent; however, the restoration of FXN levels in patient cells provides an obvious therapeutic strategy for the disease. Bone marrow-derived hematopoietic stem cells, with their capacity for self-renewal and ability to penetrate many organs of the body following their differentiation into mature leukocytes, may provide a potential means of delivering the much-needed FXN protein to affected tissues for an extended period. Indeed, previous studies by Rocca et al. and Kemp et al. observed that the transplantation of allogeneic wild-type mouse hematopoietic stem and progenitor cells (HSPCs) into the YG8sR mouse model of FRDA enabled the delivery of FXN to the brains and spinal cords of transplant recipient mice and partially ameliorated disease symptoms.^6^^,^^7^ Such findings indicate the potential of HSPC transplantation as a form of FXN replacement therapy for patients with FRDA. However, a potential limitation of such a treatment is that transplanted, non-FRDA stem cells may be incapable of fully restoring FXN levels in affected individuals, leading to partial therapeutic effects, as seen in the studies mentioned above. A more pressing issue that greatly hampers the wider use of HSPC transplantation therapy is the relatively high mortality rate resulting from graft-versus-host disease (GvHD) post treatment^8^ and the subsequent need for histocompatible human leukocyte antigen (HLA)-matched bone marrow (BM) donors.^9^ The use of the patient’s own stem cells for autologous transplantation provides an obvious solution to the problem of transplant rejection and GvHD,^10^^,^^11^ while the challenge of ensuring sufficient replenishment of FXN in FRDA cells may be resolved by generating genetically engineered blood stem cells that can secrete FXN protein capable of penetrating target cells and rescuing their function. To this end, we fused a signal peptide and a cell-penetrating sequence from either the HIV transactivator of transcription (TAT) peptide, the prototype cell-penetrating peptide, or from human annexin-3, to the amino terminus of the full-length FXN precursor protein (1–210 amino acid). The latter peptide, if functional, should be preferable to the viral TAT sequence to avoid potential adverse immune reactions. The rationale of the design was to obtain a secreted version of FXN that can be released into the environment by modified cells and penetrate target tissues. Once internalized, the fusion protein containing the full-length form of human FXN (23 kD) is processed in the mitochondria by peptidases, which release the mature, biologically active form (81–210 amino acids, 14 kD),^12^^,^^13^ separating it from the secretion and penetration sequences. We validated this strategy by subcloning the cDNA encoding the engineered FXN into lentiviral vectors, which were used in FRDA models *in vitro* and *in vivo.*

## Results

### Generation of secreted, cell-penetrating FXN proteins with functional activity

**Figure 1:**
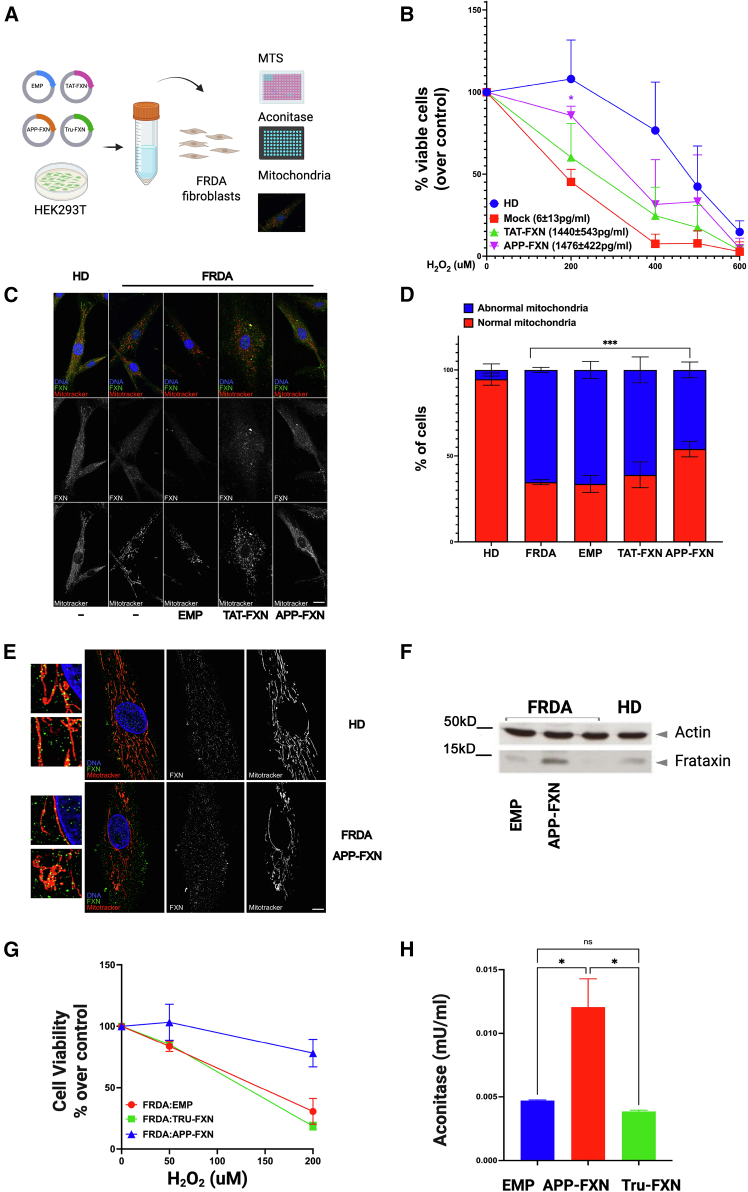
Functional validations of secreted and cell-penetrating frataxin proteins (A and B) (A) Cartoon summarizing the scheme of the functional experiments; (B) healthy donor (HD) (*n* = 3) and FRDA fibroblasts were exposed to supernatants from polyethylenimine (PEI)-only, TAT-FXN, or APP-FXN plasmid-transfected HEK293 cells (*n* = 4) and analyzed for viability post H_2_O_2_ treatment; mean values ± SEM are shown; ∗*p* < 0.05, multiple comparison two-way ANOVA with Bonferroni’s post hoc test. (C) Representative images of HD and FRDA fibroblasts stained for FXN (green) and counterstained with 4′,6-diamidino-2-phenylindole (DAPI) (blue) and mitotracker (red). FRDA fibroblasts were treated with supernatants from empty (EMP) plasmid, TAT-FXN, and APP-FXN-transfected HEK293T cell cultures, compared with HD and untreated FRDA fibroblasts. Scale bars, 20 μm. (D and E) (D) Quantification of cells with abnormal mitochondria from the experiment shown in (C). The values represent the means of 3 independent biological replicas. Sample size: HD = 200, FRDA = 193, FRDA-EMP = 206, FRDA-TAT-FXN = 228, and FRDA-APP-FXN = 239. Mean values ± SEM are shown, ∗∗∗*p* < 0.001, chi-square test (E). Representative images of Deep Structured Illumination Microscopy (SIM) of FRDA fibroblasts immunostained with anti-FXN antibody (green) and counterstained with DAPI (blue) and mitotracker (red), treated with supernatants from APP-FXN-transfected HEK293T cell cultures (bottom). Normal human fibroblasts (HD) were used as control (upper panel). Scale bars, 10 μm. (F) Western blot analysis of FRDA fibroblasts showing increased levels of mature (14 kDa) FXN in cell lysates of cells exposed to supernatants from HEK293T cell cultures transfected with the APP-FXN plasmid construct compared with empty plasmid-transfected controls (EMP). HD fibroblasts were included as a positive control. The blot is representative of two separate experiments; (G) Comparison of the effects of treatment with culture supernatants from EMP, truncated (TRU)-FXN, or APP-FXN plasmid-transfected HEK293T cells on FRDA fibroblasts’ viability following H2O2 exposure. (H) Aconitase activity levels. Mean values ± SEM are shown; *N* = 2–4/group, ∗*p* < 0.05, multiple-comparison one-way ANOVA with Bonferroni’s post hoc test.

We designed modified versions of the FXN precursor protein, consisting of 210 amino acids, by fusing a secretory signal (SS) peptide and a penetration peptide derived either from the HIV TAT^14^ or human annexin-3 (APP) to its amino terminus.^15^ Codon-optimized sequences encoding wild-type FXN and the FXN fusion proteins were cloned into the lentiviral pLIG vector (Figure S1A). We first transfected HEK293T cells with the pLIG-FXN constructs (FXN and APP-FXN) to measure FXN secretion into the supernatant by ELISA (Figure S1B, left). Murine primary hematopoietic cells and a neuronal cell line were then incubated with supernatants from HEK293T cells expressing either wild-type FXN or the fusion protein to assess uptake of human FXN in a mouse cellular background. Both secretion and uptake of FXN were enhanced when using the engineered protein (Figure S1B, right). Given these results, we next compared the relative efficiency of the TAT and APP penetrating peptides. HEK293T cells were transfected with TAT-FXN and APP-FXN constructs, and FXN levels were quantified both in cell lysates by western blot (Figure S1C) and by ELISA (Figure S1D). Both assays revealed increased levels of FXN upon transfection with both engineered FXN proteins. Of note, transfected HEK293T cells displayed increased levels of both the 23 kDa immature and the 14 kDa mature form of FXN, suggesting correct processing of the precursor protein (Figure S1C). FRDA fibroblasts are abnormally sensitive to reactive oxygen species (ROS), have a deficit in aconitase activity, and have abnormal mitochondria.^16^^,^^17^^,^^18^ To assess whether cell-penetrating FXN could rescue defects in FRDA fibroblasts, we used supernatants from HEK293T cells transfected with the different FXN constructs (Figure 1A). Fibroblasts treated with supernatants containing APP-FXN were partially protected from H_2_O_2_-induced death, behaving similarly to fibroblasts from healthy donors. The supernatant of HEK293T cells used in this set of experiments contained similar levels of FXN (TAT-FXN: 1440 ± 543 pg/mL, APP-FXN: 1476 ± 422 pg/mL), ruling out a concentration-dependent effect (Figure 1B). Cells isolated from patients with FRDA display abnormal mitochondria.^19^ The mitochondria in FRDA fibroblasts had a fragmented morphology compared with those from healthy donors, as previously reported.^20^ Treatment with supernatants containing APP-FXN partially reversed this altered phenotype (Figures 1C and 1D), with APP-FXN showing greater effectiveness than TAT-FXN. This outcome led us to select the APP-based penetrating peptide for subsequent experiments. As expected, immunofluorescence staining, confocal microscopy, and super-resolution microscopy revealed that exogenous FXN localized to mitochondria, suggesting that the fusion protein could reach its physiological target within the cell (Figure 1E). Western blot confirmed that the APP-FXN protein was incorporated and correctly processed in FRDA fibroblasts (Figure 1F). Finally, to demonstrate that the protective effect was caused by the FXN sequence and not the penetration or secretion peptides, we used a vector encoding the secretion and penetration peptides fused to a short segment in the amino-terminal region of FXN. Full length, but not truncated, FXN was able to rescue ROS-induced cell death, demonstrating that FXN, but not the secretory signal or APP peptides, is required for protection from cell death (Figure 1G). Deletion of the *Frataxin* gene has been associated with accumulation of mitochondrial iron deposits and reduced Fe-S cluster levels,^21^ resulting in deficiencies in the Fe-S cluster-containing aconitase mitochondrial enzyme.^18^^,^^22^^,^^23^ Exposure of FRDA fibroblasts to APP-FXN-containing supernatants significantly increased aconitase activity. Supernatants containing the peptide with truncated FXN was indistinguishable from control supernatants, indicating specificity of the effect (Figure 1H).

### Lentiviral expression of secreted frataxin in normal or FRDA-derived CD34^+^ progenitor cells does not perturb hematopoietic differentiation *in vitro*

**Figure 2:**
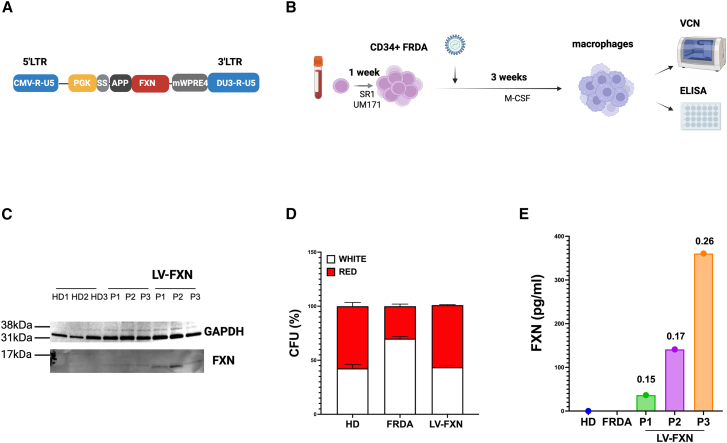
Differentiation of CD34^+^ hematopoietic progenitor cells from patients with FRDA after transduction with a lentivirus expressing the APP-FXN fusion protein (A) Schematic representation of the pCCL-phosphoglycerate kinase (PGK)-FXN transfer vector. Cytomegalovirus (CMV) promoter, R and U5 components of HIV-1 5′ long terminal repeat (LTR); PGK promoter; secretory signal petide (SS); annexin 3 derived penetration peptide (APP); human frataxin coding sequence (FXN); WPRE4, woodchuck hepatitis virus post-transcriptional regulatory element mut 4; DU3-RU5, self-inactivating 3′ LTR. (B–E) (B) Overview of the experiment; (C) Western blotting to detect expression of frataxin (FXN) in cell lysates of normal donor (HD) or patient (P) cells, unmodified or modified (LV-FXN) by the lentivirus, 3 days after transduction. A glyceraldehyde 3-phosphate dehydrogenase (GAPDH) antibody was used as a loading control; (D) Percentage of CFU colonies (white and red colonies) in samples from healthy donors (HD) and patients with Friedreich’s ataxia (FRDA) untreated or treated (P1, P2, P3) with the frataxin-containing vector (LV-FXN); (E) frataxin secreted into the medium by FRDA CD34^+^ derived macrophages, untransduced or transduced with the frataxin lentivirus, was detected by ELISA. Numbers on top of the bars indicate average vector copy numbers by droplet digital PCR.

Before embarking on transplantation experiments in a mouse, we wanted to assess whether expression of the FXN fusion proteins in human CD34^+^ HSPCs could disturb hematopoietic differentiation, which would be undesirable in view of potential clinical trials. Thus, we transduced CD34^+^ HSPCs with the pLIG virus expressing the TAT- or APP-FXN variants, and after 72 h we quantified protein expression by western blotting. Results showed higher levels of FXN in cells transduced with virus encoding the FXN fusion proteins compared with those treated with control virus. Notably, western blot assessment of culture supernatants from the same HSPCs revealed that cells transduced with the FXN fusion sequence secreted substantial levels of the immature FXN fusion protein (Figure S2A). Subsequently, the HSPCs were cultured in semisolid media for a 2-week period, and the number of red/white colonies generated from CD34^+^ HSPCs transduced with either lentivirus containing the empty vector, the TAT-FXN vector, or the APP-FXN vector was scored by microscopy analysis. There was no difference in the number of colonies derived from the FXN-encoding vectors compared with control lentivirus (Figure S2B). We next cloned the cDNA encoding APP-FXN into a clinical-grade vector (pCCL), currently used in phase I and II clinical trials, and packaged the construct into a lentiviral vector delivery system.^24^^,^^25^^,^^26^^,^^27^^,^^28^ The clinical FXN vector is hereafter referred to as LV-FXN (Figure 2A). We used this virus to transduce CD34^+^ HSPCs donated by three patients with FRDA (Table S1; a cartoon illustrating the experimental steps is shown in Figure 2B). After transduction, HSPCs were induced to differentiate *in vitro*, and expression of exogenous FXN in cell lysates from healthy donors or patients with FRDA was detected by western blotting (Figure 2C). Colony forming units (CFUs) were scored after 2 weeks. The number of white or red blood colonies obtained from the lentivirally transduced FRDA cells was similar to that of cells derived from an untreated normal donor, suggesting that the vector had no negative impact on hematopoietic differentiation *in vitro* (Figure 2D). Notably, myeloid cells that received the gene therapy, but not unmodified normal or FRDA cells, secreted FXN protein into the culture supernatant at levels proportional to vector copy number (Figure 2E).

### Autologous transplantation of HSPCs modified with a lentiviral vector causes secretion of the cell-penetrating frataxin protein in the blood of YG8sR FRDA mice

**Figure 3:**
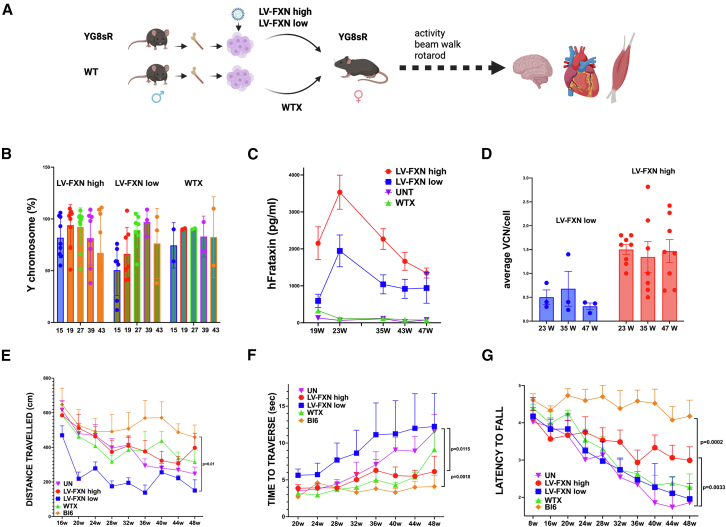
YG8sR mice transplanted with HSPCs transduced with the APP-FXN lentiviral vector display increased blood FXN levels in recipients and delayed onset of motor symptoms (A–G) (A) Female FRDA mice were transplanted at 8 weeks of age with male HSPCs lentivirally transduced with LV-FXN at low (LV-FXN low) and high dose (LV-FXN high) and assessed until 48 weeks of age for effects of treatment on motor symptoms and disease-affected tissues; (B) male Y-chromosome PCR analysis in white blood cells of female recipients to determine the percentage of donor HSPC engraftment at different times post-transplantation; (C) ELISA analysis of mean (±SEM) human FXN levels in the blood of HSPC recipient FRDA mice and untreated controls post-transplant; (D) average vector copy number (VCN) in leukocytes of mice transplanted with LV-FXN (high or low dose) transduced HSPCs at different times post-transplantation; (E) mean (±SEM) activity levels indicated by distance traveled over 4 min; (F) balance/coordination assessed by beam walk test, as well as by rotarod test (G), in transplant recipient FRDA mice and untransplanted YG8sR and C57BL/6 controls up to 48 weeks of age. LV-FXN low transduced HSPC transplanted mice (*N* = 3–4), LV-FXN high transduced HSPC-transplanted mice (*N* = 8), WTX = C57BL/6 HSPC transplanted mice (*N* = 5), UN = untransplanted YG8sR (*N* = 8–9), and BL6 = untransplanted wild-type (*N* = 5) controls. *p* values indicate significance after simple linear regression.

We hypothesized that transplantation of genetically modified HSPCs could provide a long-term delivery vehicle for functional FXN to increase endogenous cellular FXN levels and ultimately inhibit disease progression and reduce symptoms. BM cells were isolated from the femurs and tibias of male FRDA mice (YG8sR[GAA] > 700)^29^ and lineage-negative (Lin−) HSPCs purified by magnetic cell sorting (to a purity of ∼60% as assessed by flow cytometric analysis for surface c-Kit expression), transduced with the LV-FXN lentivirus at ∼6 MOI (LV-FXN low) or ∼12.5 MOI (LV-FXN high), and then transplanted into pre-symptomatic, 8-week-old female FRDA mice. Physiological levels of expression of the FXN isoforms by the lentiviral vector were validated by western blotting of transduced Lin− cells (Figure S3). Since transplantation of unmodified wild type HSPCs has been shown to ameliorate symptoms in the YG8sR model,^6^^,^^7^ we also included a group of YG8sR female mice receiving wild-type HSPCs from syngeneic C57BL/6 male donors (WTX). A cartoon illustrating the different steps of the experiment is shown in Figure 3A. After transplantations, successful engraftment of male donors in female recipients was assessed by periodic bleedings, and purified white blood cells DNA was subjected to real-time PCR analysis for the detection of male testis-specific protein (*TSPY*) gene on the Y chromosome. TSPY levels revealed that all but one of the recipient mice receiving lentivirally transduced HSPCs had been reconstituted with male, *TSPY* gene-expressing donor HSPCs, averaging 80%, by 39–43 weeks post-transplantation (Figure 3B). To confirm production and secretion of the FXN fusion protein by the transplanted HSPCs, blood samples from mice at different time points were subjected to ELISA. The highest levels of FXN were detected in the plasma of mice receiving HSPCs transduced with a high dose of LV-FXN lentivirus (LV-FXN high), followed by mice transplanted with HSPCs infected with low dose virus (LV-FXN low). Mice transplanted with C57BL/6 HSPCs (WTX), which do not secrete exogenous human FXN, as well as the untransplanted YG8sR control mice (UN), had negligible levels of FXN detected in their blood (Figure 3C). Vector copy number analysis detected around 0.5 viral copies per cell in the low-dose transduced group, whereas around 1.5 copies per cell were detected in the high-dose group (Figure 3D). A gradual decrease in blood FXN was observed from 23 weeks post-transplantation in the majority of mice. This was not caused by gradual loss of transplanted cells, as indicated by the high percentage of Y chromosome reconstitution after 43 weeks (Figure 3B) and normal blood leukocyte cell counts in recipient mice (Table S2). An alternative explanation may be that gene therapy-modified HSPCs are unstable and have been outgrown by the untransduced fraction of transplanted HSPCs. VCN analysis confirmed that proviral DNA levels in blood leukocytes of recipient mice in both the APP-FXN high and low groups remained relatively stable between 23 to 47 weeks of age, indicating that there was no selective reduction of APP-FXN-positive HSPC/leukocyte populations in these transplant recipients (Figure 3D). The most plausible explanation for this fluctuation is the establishment of output from a more stem-like population once the wave of progenitors fades.

### Delayed motor deficits in YG8sR mice transplanted with HSPCs secreting cell-penetrating frataxin

We tested the motor function of transplanted mice up to the age of 48 weeks, i.e., 40 weeks post HSPC transplantation. The activity, beam walk, and rotarod tests were undertaken prior to treatment at 8 weeks of age, then 8 weeks post-transplantation, and every 4 weeks thereafter. Previous studies have observed age-related declines in YG8sR mouse performance in these tests compared with non-diseased C57BL/6 age-matched control mice.^30^ Activity tests highlighted statistically significant inter-group differences only between normal untransplanted BL6 and transplanted LV-FXNlow mice, which actually performed worse than controls, perhaps due to the procedure. This result may suggest that the activity test is not sufficiently challenging to induce deterioration in these FRDA mice and may eventually have shown benefits of the therapy if the test had been performed beyond 48 weeks of age (Figure 3E). The beam walk balance test results revealed significant (*p* = 0.0018) age-related performance declines in untreated FRDA mice versus wild-type controls, which became evident from approximately 36 weeks of age onwards. FRDA mice transplanted with LV-FXNhigh HSPCs, however, did not show declines in beam walk performance compared with C57BL/6 controls and, more importantly, were significantly (*p* = 0.0115) better at traversing the beam compared with the untransplanted group, indicating a beneficial effect of the therapy on motor function. LV-FXN low HSPCs showed no significant difference in performance compared with the untransplanted group. The beneficial effects of wild-type HSPCs transplantation on beam walk performance were also noted, as reported previously^6^^,^^7^ (Figure 3F).

Finally, motor co-ordination and skeletal muscle efficiency analysis by rotarod testing revealed significant (*p* = 0.0002) decreases in performance in the untreated FRDA mice compared with healthy controls from 24 weeks of age. Notably, the LV-FXN high HSPC-transplanted mice performed significantly better than untransplanted mice in the rotarod assay (*p* = 0.0033), further indicating the beneficial effect of genetically modified HSPC transplantation on FRDA mouse motor function (Figure 3G). Similar to the beam walk test, no beneficial effects were seen with transplantation of HSPCs expressing low levels of secreted FXN, indicating the requirement for a minimal level of restoration for benefits on motor function to be achieved.

### Anatomical and biochemical changes in YG8sR mice transplanted with HSPCs secreting cell-penetrating frataxin

**Figure 4:**
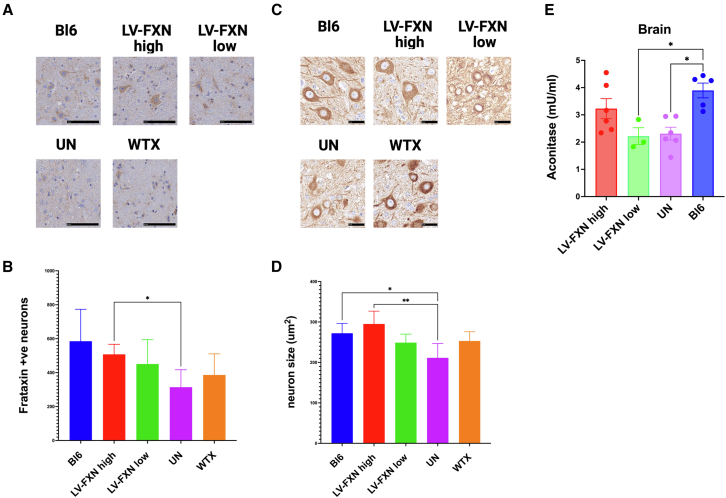
Transplantation of HSPCs transduced with the APP-FXN secreting lentivirus into YG8sR mice prevents size reductions of dentate nucleus resident neurons in the cerebellum and increases aconitase activity (A–E) (A) Cerebellar dentate nucleus neurons were stained with a frataxin antibody; scale bars, 100 μm; quantification of staining in Bl6 (*n* = 4), LV-FXN high (*n* = 5), LV-FXN low (*n* = 3), UN, and WTX (*n* = 4) is shown in (B) as mean ± SEM; (C) beta-III tubulin staining of dentate nucleus resident neurons, scale bars, 25 μm. (D) Mean surface area (±SEM) of dentate nucleus neurons in Bl6 (*n* = 3), LV-FXN high (*n* = 5), LV-FXN low (*n* = 3), UN (*n* = 4), and WTX (*n* = 4) mice; (E) Mean aconitase activity (±SEM) in the brain of transplanted mice; untransplanted (UNT) *n* = 6, LV-FXN high *n* = 6, and LV-FXN low *n* = 3. ∗*p* < 0.05,∗∗*p* < 0.01; multiple-comparison one-way ANOVA test with Bonferroni’s post hoc analysis.

It has been shown previously that the number of FXN-positive cerebellar dentate nucleus neurons is decreased in an inducible mouse model of FRDA.^31^ Immunohistochemical analysis of dentate neurons confirmed that there was a drastic decrease in FXN-positive dentate neurons in YG8sR mice compared with isogenic control C57BL6 mice. Notably, there was a significant increase in FXN-positive neurons in mice transplanted with HSPCs transduced with high dose of LV-FXN virus compared with untransplanted mice. In keeping with previous results,^7^ mice transplanted with unmodified wild-type HSPCs also showed increased numbers of cerebellar neurons positive for FXN, although in this case the difference was not statistically significant (Figures 4A and 4B).

**Figure 5:**
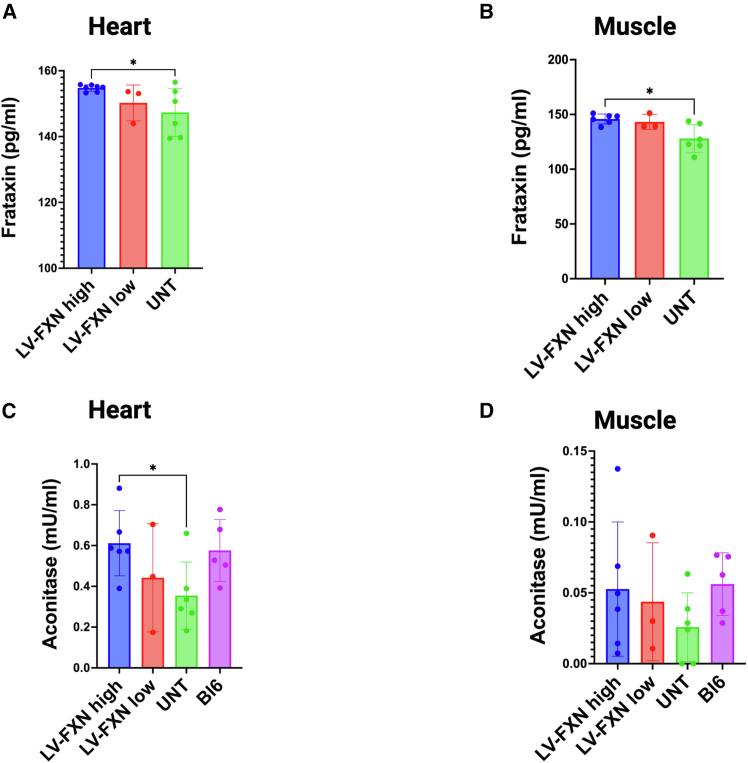
Transplantation of APP-FXN fusion protein secreting HSPCs into YG8sR mice increases frataxin protein and aconitase activity in the hearts and muscles of YG8sR mice Quantification of human frataxin in hearths (A) and muscles (B) in the different treatment groups was carried out by ELISA (UN *n* = 6, LV-FXN high *n* = 6–7, and LV-FXN low *n* = 3); ∗*p* < 0.05, multiple-comparisons one-way ANOVA test with Bonferroni post hoc. Mean aconitase activity in hearts (C), ∗*p* < 0.05, multiple-comparisons one-way ANOVA test with Scheffé's post hoc, and muscle (D) tissue homogenates for each mouse treatment group.

**Figure 6:**
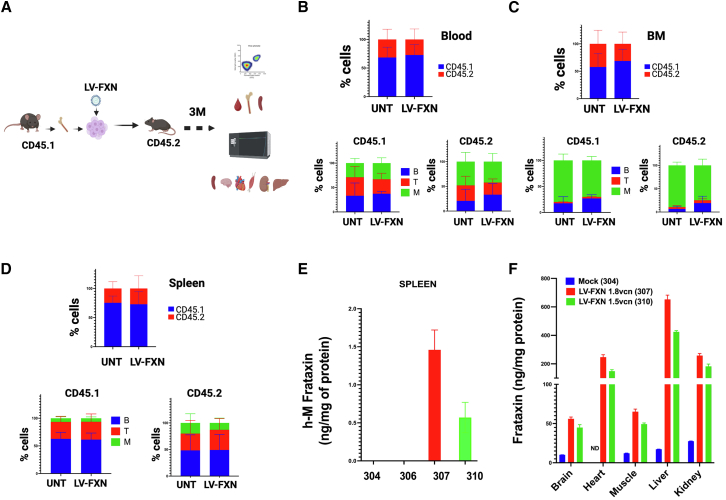
LV-FXN gene therapy does not affect the engraftment and lineage commitment of HSPCs and deposits FXN protein in FRDA-relevant tissues (A) Overview of the transplantation experiment. Lineage-negative cells isolated from LY5.1 mice were transduced with LV-FXN at a multiplicity of infection (MOI) of 20 and transplanted into lethally irradiated LY5.2 recipient mice. Three months after transplantation, hematopoietic organs were analyzed by FACS, and frataxin protein levels were measured in the spleen, brain, heart, muscle, liver, and kidney by mass spectrometry. (B–D) Percentage of CD45.1 (donor-derived) and CD45.2 (recipient-derived) cells in peripheral blood, bone marrow (BM), and spleen of mice transplanted with mock-untransduced (*n* = 3) or LV-FXN-transduced cells (*n* = 4) (upper), along with the lineage composition within the CD45.1 and CD45.2 compartments (lower). (E) Levels of human mature frataxin (ng per mg of total protein; mean ± SD) in the spleen of mice transplanted with mock-untransduced cells (mouse #304) or LV-FXN-transduced cells (mice #306, #307, and #310). (F) Total frataxin levels (ng per mg of total protein; mean ± SD) in the indicated organs of mice transplanted with mock-transduced cells (mouse #304) or LV-FXN- transduced cells with >1 vector copy number (VCN) (mice #307 and #310). ND = not determined.

Lack of FXN causes progressive atrophy of the dentate nucleus in patients with FRDA.^32^ In order to detect morphological changes in dentate nucleus neurons before and after gene therapy, we stained cerebellar sections of FRDA mice with betaIII-tubulin at 48 weeks of age. As expected, microscopic analysis of the cerebellar dentate nucleus showed significant reductions in the size of neurons in FRDA (YG8sR) mice versus wild-type (C57BL6) controls. In contrast, the size of neurons in FRDA mice transplanted with HSPCs transduced with the high-dose LV-FXN lentivirus was similar to that of wild-type mice and ∼35% larger than that of untransplanted controls (Figures 4C and 4D). Cerebellar neurons of FRDA mice transplanted with low-dose LV-FXN or wild-type HSPCs also showed approximately a 25% increase in size, although this was not statistically significant. The iron-sulfur cluster-dependent enzyme aconitase is less active in FRDA, leading to reduced enzyme function in both patients and FRDA models.^33^^,^^34^ In the brain, transplantation of HSPCs transduced with highdose, but not lowdose, LV-FXN virus increased aconitase activity in transplanted versus untransplanted FRDA mice, although the difference did not reach statistical significance (Figure 4E). Other tissues relevant to the pathology of FRDA are the heart and muscle.^35^^,^^36^^,^^37^ First, we detected an increase in human FXN expression in the heart and skeletal muscle of transplanted mice vs*.* untransplanted controls, which was proportional to the virus dosage (Figures 5A and 5B). The activity of aconitase paralleled FXN protein levels, suggesting that restoration of FXN levels by the gene therapy also rescued biochemical function in these organs (Figures 5C and 5D). Concerns have been raised about FXN overexpression and associated toxicity, as reported in a recent AAV-based study.^38^ Using a congenic mouse model, we assessed whether our gene therapy approach affects HSPC engraftment or lineage distribution across hematopoietic organs (Figure 6A; Figure S4). We observed no significant differences in lineage composition (Figures 6B–6D). To evaluate human mature FXN in non-hematopoietic tissues, we performed mass spectrometry. Using the human-specific peptide, mature FXN (hFXN-M) was detectable only in spleen samples from two gene-therapy-treated mice that had >1 vector copy per cell (Figure 6E). Lack of hFXN-M in other organs could be due to concentrations below the detection limit of the species-specific assay. Analysis of the distribution of total—mouse plus human—FXN in two gene therapy-treated mice across non-hematopoietic organs (brain, liver, heart, muscle, and kidney) demonstrates that total FXN levels are elevated compared with controls in all tissues (Figure 6F).

## Discussion

Currently, an effective therapy for FRDA remains elusive. A number of therapeutic approaches for FRDA have been investigated, including strategies that promote increased FXN levels,^39^^,^^40^^,^^41^ reducing oxidative stress^42^^,^^43^ and prevention of cellular iron accumulation.^42^ However, the difficulty of getting therapeutic agents into the CNS, across the blood-brain barrier (BBB), poses a major obstacle to the effectiveness of many of these factors.^44^^,^^45^ Cell therapy has shown potential in mouse models of FRDA. Transplantation of wild-type HSPCs into YG8sR FRDA mice displayed therapeutic effects on motor coordination deficits, muscle weakness, and degeneration of sensory neurons. Mechanistically, HSPCs differentiated into microglia and macrophages, delivering FXN protein via cell-cell bridges.^6^^,^^7^ Indeed, microglia can even transfer healthy mitochondria, rescuing neurons from oxidative stress and dysfunction via tunneling nanotubes.^46^ HSPCs that develop into circulating monocytes can migrate across the BBB and enter the brain to differentiate into resident microglia, thereby repopulating this cell population following their depletion.^47^^,^^48^^,^^49^ Such a capacity of HSPCs provides a means of potentially delivering therapeutic agents into the brain, using these cells as transporters for such factors. We attempted to enhance the observed beneficial effects of HSPC transplantation by modifying these cells to continuously secrete a cell-penetrating FXN fusion protein, with the aim of allowing a more efficient replenishment of FXN within target cells. Furthermore, autologous rather than allogeneic stem cell transplantation should resolve the significant challenge posed by transplant rejection when using a nonperfectly HLA-matched donor, reducing the high mortality risk from GvHD complications in transplant recipients.^50^^,^^51^ Following transplantation, we observed efficient engraftment of donor cells within hosts at least 30 weeks after transplantation, with high levels of human FXN in the blood of YG8sR mice treated with HSPCs producing the FXN fusion protein, but not in the control groups, indicating that donor cells had engrafted and secreted the APP-FXN fusion protein. However, we noted that elevations in blood FXN were not constant in recipient mice, decreasing gradually from 15 weeks after HSPC transplantation. Although, it is possible that the therapeutic effects of donor HSPCs would gradually wane as their numbers within recipients diminish, or their capacity to secrete therapeutic protein decreases over time, an ability for these modified HSPCs to impact disease symptoms, as well as anatomical and biochemical changes, was nonetheless apparent at least up to 40 weeks after transplantation. The composition of blood cells also changes during the first months of reconstitution, with myeloid cells initially populating the blood, followed later by the lymphoid compartment.^52^ While this could affect the amount of FXN in blood, it should not impact the rescue of brain symptoms, as macrophage-derived microglia replace resident cells relatively soon after the conditioning injury. In a clinical setting, the use of busulfan as a conditioning agent will facilitate this process.^47^

It has been shown previously that acute overexpression of FXN achieved via intravenous injections of engineered adeno-associated virus (AAV) can lead to cardiac or hepatic toxicity in mice.^38^^,^^53^ We did not detect increased mortality in mice transplanted with highdose LV-FXNtransduced HSPCs compared with other transplantation groups. Blood counts in YG8sR mice, as well as hematopoietic differentiation of human CD34^+^ cells from a normal donor or an patient with FRDA, did not show a negative impact of the lentivirally expressed FXN fusion protein (Figures 3 and 6; Table S2). The lentiviral construct restored FXN levels in target tissues of transplanted mice, such as heart, brain, and muscle, at near physiological levels (Figures 4 and 5), suggesting that the lentiviral delivery approach should also be safe for patients. Safety of the approach is corroborated by experiments in a congenic mouse model, in which transplantation of LV-FXN-modified HSPCs reconstituted the hematopoietic system without lineage distortion (Figures 6A–6D). A dedicated biodistribution study, including multiple lentiviral vector doses and a good manufacturing practice (GMP)-like vector, will be required prior to a clinical trial application.

In a recent study, a synthetic FXN-TAT fusion peptide was purified and injected into mice with an FRDA-like disease, increasing their lifespan.^54^ Larimar Therapeutics, a US-based biotech company, has completed a placebo controlled phase 2 study in patients with FRDA dosed with subcutaneous injections of the FXN-TAT fusion peptide (nomlabofusp). The drug was generally well tolerated and demonstrated dose-dependent increases in FXN levels in all evaluated tissues (skin and buccal cells) after daily dosing of 14 days, followed by every-other-day dosing until day 28 in the 25 and 50 mg cohorts (https://investors.larimartx.com/news-releases/news-release-details/larimar-therapeutics-reports-positive-top-line-data-phase-2-dose). Compared with this strategy, our cell and gene therapy method presents several advantages: (1) there is no risk of acute toxicity posed by systemic injections of high concentrations of the synthetic peptide; (2) delivery of FXN would be continuous and long term; and (3) fusing FXN to a penetrating peptide of human origin (APP) should minimize the risk of potentially dangerous immune reactions and/or transplanted cells rejection. *Ex vivo* gene therapy approaches are currently being used in clinics for several metabolic disorders,^55^ including metachromatic leukodystrophy, which has now received approval from the US Food and Drug Administration (FDA) and the Medicines and Healthcare products Regulatory Agency (MHRA) under the name Libmeldy.^56^ Therefore, our approach is likely to be safer, more long-lasting, and more cost-effective than systemic drug injections that would be required throughout the patient’s entire lifetime.

### Limitations of the study

There are several limitations of the study. First, sensitivity to ROS-induced killing was highly variable in patient- and volunteer-derived fibroblasts, necessitating selective testing approaches. Second, the therapeutic benefits of the strategy were observed in a single genetic model of FRDA, mainly used to capture motor and balance coordination deficits. Additional mouse models will be needed to assess the efficacy of the therapeutic approach in prolonging survival and supporting organ systems, such as the heart, that are affected by lack of FXN.

## Resource availability

### Lead contact

Further information and requests for resources and reagents should be directed to and will be fulfilled by the lead contact, Arturo Sala (arturo.sala@brunel.ac.uk).

### Materials availability

Plasmids generated in this study will be available on request upon completion of a material transfer agreement.

### Data and code availability

• All data are available upon reasonable request to the lead contact, Arturo Sala (arturo.sala@brunel.ac.uk).
• No custom code was generated in this study.
• Any additional information required to reanalyze the data reported in this paper is available from the lead contact upon request.

## Acknowledgments

We thank Paola Vagnerelli for assistance and expertise with the microscopic analysis of cells; Robert Spaull for collection of patient samples; and Marta Zinicola and Andrea Schejtman for initial testing of the frataxin construct; and Elizabeth McCarthy (Microscopy facility manager, Brunel) for support with super-resolution microscopy. This project has received funding from the Medical Research Council (MRC) and Friedreich’s Ataxia Research Alliance (FARA) grants to A.S. The work was supported in part by the Wellcome Trust, (217112/Z/19/Z), the Great Ormond Street Hospital Children’s Charity, and the UCL Technology Fund. A.J.T., B.J.C., G.S., S.E.M., and T.E.W. were also supported by the National Institute for Health and Care Research Biomedical Research Centre at Great Ormond Street Hospital for Children National Health Service Foundation Trust and University College London. The following figures were created in BioRender: graphical abstract, Figure 1 (https://biorender.com/6hoex22), Figure 2 (https://biorender.com/4fu3v4s), Figure 3 (https://biorender.com/gfjy6gx), Figure 6 (https://biorender.com/mr3g5wk), and Figure S1 (https://biorender.com/co1zanf)

## Author contributions

Conceptualization, A.S. and G.S.; methodology, J.P.-L., E.S., S.E.M., B.J.C., T.E.W., S.S., S.A.-V., E.K.-E., S.E.M., K.S, I.A.B., and T.R.; investigation, J.P.-L., E.S., S.E.M., and K.S.; supervision, A.S., G.S., and A.J.T.; patient samples collection, M.A.K., L.C. and J.H; writing – original draft, J.P. and A.S.

A.S. and G.S. jointly led this study.

## Declaration of interests

The codon-optimized DNA sequence encoding the fusion protein described in the manuscript has been the subject of UK Patent application no. 2413430.6.

A.S. and Brunel University London.

## STAR★Methods

### Key resources table

**Table:** 

REAGENT or RESOURCE	SOURCE	IDENTIFIER
**Antibodies**		

anti-FXN recombinant rabbit monoclonal antibody	Abcam, Waltham, MA	17A11, Ab113691; RRID:AB_10862125
Anti-mouse CD11B APC Cy7	Biolegend	Cat# 101226; RRID:AB_830642
Anti-mouse CD3 SBV440	Biorad	Cat# 64599646
Anti-mouse CD45.1 PE	Miltenyi	Cat# 130-102-499; RRID:AB_2660704
Anti-mouse CD45.2 FITC	Miltenyi	Cat# 130-102-458; RRID:AB_2660717
Anti-mouse CD45R APC	Miltenyi	Cat# 130-123-569; RIDD:AB_2819495
Donkey polyclonal anti-Mouse FITC	Jackson ImmunoResearch Labs	Cat# 715-096-150, RRID:AB_2340795
GAPDH mouse monoclonal IgG antibody	Santa Cruz Biotechnology	sc-166574; RRID:AB_2107296
Mouse anti-actin (C-2)	Santa Cruz	Cat#sc-8432; RRID:AB:626630
Mouse anti-beta III tubulin [2G10]	Abcam	Cat# ab78078; RRID:AB_2256751
Mouse anti-GAPDH (1E6D9)	Proteintech	Cat# 60004-1-Ig; RRID:AB_2107436
Mouse anti-human frataxin (18A5DB1)	Abcam	Cat# ab110328; RRID:AB_10866539
Mouse anti-mouse CD19-FITC (QA17A27)	Biolegend	Cat# 159807; RRID:AB_2876560
Mouse monoclonal anti-frataxin (18A5DB1)	Abcam	Cat# ab110328 RRID:AB_10866539
Rabbit anti-frataxin [EPR21840]	Abcam	Cat# ab219414
Rat anti-mouse CD11b-APC (M1/70)	Biolegend	Cat# 101211; RRID:AB_312794
Rat anti-mouse CD3-pacific blue (17A2)	Biolegend	Cat# 100213; RRID:AB_493644
Rat anti-mouse cKit-FITC (2B8)	Biolegend	Cat# 105805; RRID:AB_313214
Rat Anti-mouse/human CD11b-APC (M1/70)	ThermoFisher Scientific	Cat # 17-0112-82; RRID:AB_469343

**Bacterial and virus strains**		

NEB® Stable Competent *E. coli* (High Efficiency)	New England Biolabs	C3040H

**Biological samples**		

Gibco Fetal Bovine Serum	ThermoFisher Scientific	Cat# A5256701
Human mobilised Leukopak	AllCells, Alameda, CA, USA	Cat# mLPReg F
Human fibroblasts (from healthy and FRDA patients)	Dr. Mark Pook, Dr. Sara Anjomani Virmouni	N/A
Invitrogen Normal Goat Serum	ThermoFisher Scientific	Cat# 31873
Trypsin protease modified sequencing grade	Promega Corporation, Madison, WI	V5111

**Chemicals, peptides, and recombinant proteins**		

4x Laemmli Sample Buffer	BioRad	Cat#1610747
Acetic acid (glacial)	MilliporeSigma, Billerica, MA	1.00066
Acetonitrile (ACN), LC/MS Grade	Fisher Scientific, Pittsburgh, PA	A955-4
ACROS Organics hydrogen superoxide (H_2_O_2_)	ThermoFisher Scientific	Cat# 302860025
Bovine serum albumin	MilliporeSigma, Billerica, MA	A7030-10G
Cell Conditioning Solution CC1	Roche	Cat#950-500
Cell Conditioning Solution CC2	Roche	Cat#950-123
Cell Titer 96(R) Aqueous One Solution Assay, 200 assays	Promega	G3582
Complete protease cocktail	MilliporeSigma, Billerica, MA	11697498001
Dimethyl pimelimidate dihydrochloride (DMP)	MilliporeSigma, Billerica, MA	D8388
Direct Lineage Cell Depletion Kit	Miltenyi Biotec	130-110-470
Formic acid, Optima LC/MS	ThermoFisher Scientific, Waltham, MA	A117-50
Frataxin-M protein standard (50 ng in 10μL)	Blair Lab, Philadelphia, PA	Anal Chem 2018;90:2116
Gibco Dulbecco’s Modified Eagle Medium (DMEM)	ThermoFisher Scientific	Cat#11965092
Gibco RPMI 1640 Medium	ThermoFisher Scientific	Cat# 11875093
Gibco™ human M-CSF Recombinant Protein Peprotech	ThermoFisher Scientific	Cat# 300-25-2UG
Human Flt-3 Ligand (FLT3L) Recombinant Protein, PeproTech	ThermoFisher Scientific	Cat# 300-19-2UG
Human Hematopoietic Stem Cell Expansion Cytokine Package (Flt3 ligand, SCF, TPO, IL3) Peprotech	ThermoFisher Scientific	HHSC3
Human IL-3 Recombinant Protein, PeproTech	ThermoFisher Scientific	Cat#200-03-10UG
Human IL-6 Recombinant Protein, PeproTech	ThermoFisher Scientific	Cat#200-06-20UG
Human SCF Recombinant Protein, PeproTech	ThermoFisher Scientific	Cat# 300-07-2UG
Human TPO (Thrombopoietin) Recombinant Protein, PeproTech	ThermoFisher Scientific	Cat# 300-18-2UG
Hydrogen peroxide solution	Fisher Scientific UK	CAS Number-7732-18-5, 7722-84-1, 1 L, −50.00%
Invitrogen MitoTracker Red CMXRos	ThermoFisher Scientific	Cat# M46752
LentiBOOST^TM^ GMP grade-	Revvity Gene Delivery GmbH	cat# SB-A-LF-902-02
Methanol, Optimal LC/MS Grade	Fisher Scientific, Pittsburgh, PA	A454-4
Mouse Flt-3 Ligand (FLT3L) Recombinant Protein, PeproTech	ThermoFisher Scientific	Cat# 250-31L-2UG
Mouse IL-3 Recombinant Protein, PeproTech	ThermoFisher Scientific	Cat# 213-13-2UG
Mouse SCF Recombinant Protein, PeproTech	ThermoFisher Scientific	Cat# 250-03-2UG
Mouse TPO (Thrombopoietin) Recombinant Protein, PeproTech	ThermoFisher Scientific	Cat# 315-14-2UG
Paraformaldehyde	ThermoFisher Scientific	Cat# 416785000
Penicillin-Streptomycin	Sigma Aldrich	Cat# P4333
Polyethylenimine	Sigma Aldrich	Cat#408727
Protamine sulfate	ThermoFisher Scientific	Cat# J62926.06
RIPA lysis buffer with EDTA	ThermoFisher Scientific, Waltham, MA	J61529.AP
Roche cOmplete, EDTA-free Protease Inhibitor	MeRCK	Cat#4693132001
SILAC-frataxin-M protein internal standard (50 ng in 10 μL)	Blair Lab, Philadelphia, PA	Anal Chem 2018;90:2116
Stemreginin 1	STEMCELL Technologies	Cat# 72344
StemSpan media	STEMCELL Technologies	Cat# 09650
TaqMan Fast Advanced Master Mix	Thermo Fisher	Cat#4331182
UM171	STEMCELL Technologies	72912
VECTASHIELD Antifade Mounting Medium with DAPI	Vector Laboratories	Cat# H-1200-10
Water, Optima LC/MS Grade	Fisher Scientific, Pittsburgh, PA	Cat# 600-30-76

**Critical commercial assays**		

Aconitase Assay Kit	Abcam	Cat# ab83459
Cell Titer 96(R) Aqueous One Solution Assay, 200 assays	Promega	G3582
CellTiter 96® AQ_ueous_ Non-Radioactive Cell Proliferation Assay (MTS)	Promega	Cat#G5421
Clarity Western ECL Substrate	BioRad	Cat#1705060
DAB Detection Kit for Ventana	Roche	Cat# 05266360001
ddPCR Super mix for Probes (No dUTP)	Bio-Rad	Cat# 186-3024
CD34 microBead kit, human	Miltenyi Biotec	Cat# 130-046-703
Direct Lineage Cell Depletion Kit, mouse	Miltenyi Biotec	Cat# 130-110-470
Human Frataxin ELISA Kit	Abcam	Cat# ab176112
Lipofectamine 3000 kit	ThermoFisher Scientific	Cat#L3000001
Monarch® Spin gDNA Extraction Kit	New England Biolabs	Cat# T3010L

**Experimental models: Cell lines**		

FRDA patients cell lines FA1, FA2, FA3	Dr. Aurélien Bayot (Université Paris, France);	N/A
FRDA patient cell line GM04078	Coriell Cell Repository (NJ, USA).	N/A
Healthy donor fibroblast cell line H-normal	Dr. Terry Roberts (Brunel University of London)	N/A
Healthy donor fibroblast cell lines GM23971, GM23976	Coriell Cell Repository (NJ, USA).	N/A
Human CD34^+^ HSPCs (from un-mobilised blood)	Zayed Center for Research	N/A
Human HEK293T cells	ATCC	Cat# CRL-3216
Human K562 cells	ATCC	Cat# CCL-243
Human PLB-985 cells	DSMZ	Cat# ACC 139
Lineage negative Ly 5.1 HSPCS	Zayed Center for Research	N/A
Mouse neuroblastoma NXS2	Dr. Gianluca Sala, University of Chieti-Pescara	N/A

**Experimental models: Organisms/strains**		

C57BL/6 Mouse	Charles River	C57BL/6NCrl
Mouse Y47: *Fxn*^*tm1Mkn*^ Tg(FXN)Y47Pook/J	Anjomani Virmouni et al.^57^	JAX:024097
Mouse: C57BL/6	The Jackson Laboratory	JAX:000664
Mouse: YG8sR: *Fxn*^*tm1Mkn*^ Tg(FXN)YG8Pook/2J	Kalef-Ezra et al.^58^ (Original: The Jackson Laboratory)	JAX:024113
NCI B6-Ly5.1/Cr Mouse	Charles River	B6.SJL-*Ptprc*^*a*^*Pepc*^*b*^/Boy Cr Crl

**Oligonucleotides**		

Table S3. Primers and probes used in Assessment of viral transduction efficiency and engraftment levels.	This paper	N/A
*b-actin* primer/probe set	Thermo Fisher	Cat#4331182
GAA repeat PCR phenotyping Forward 5′-AATGGATTTCCTGGCAGGACGC-3′ Reverse 5′-GCATTGGGCGATCTTGGCTTAA-3′	Kalef-Ezra et al.^58^ Anjomani Virmouni et al.^57^	N/A
TSPY primers/probe set	Thermo Fisher	Cat#4426961

**Recombinant DNA**		

5′-signal sequence truncated FXN-3′ DNA, with BamH1 restriction enzyme sites	This paper- synthesised by GeneArt (Invitrogen)	N/A
5′-signal sequence- APP-FXN-3′ DNA, with BamH1 restriction enzyme sites	This paper- synthesised by GeneArt (Invitrogen)	N/A
5′-signal sequence-TAT-FXN-3′ DNA, with BamH1 restriction enzyme sites	This paper- synthesised by GeneArt (Invitrogen)	N/A
plasmid pCMVRd8.74	Plasmid factory GmBH	PF0509
plasmid pMD.G	Plasmid factory GmBH	PF1256
Plasmids: pLIG-APP; pLIG-TAT; pLIG-TRU; pCCL-APP	This paper	N/A

**Software and algorithms**		

Biorender	Figure Labs	www.biorender.com
FACS FlowJo v10 Analyser	BD Biosciences	N/A
GraphPad Prism10	GraphPad	https://www.graphpad.com/features
Image Studio	LI-COR	https://www.licorbio.com/image-studio
ImageJ	Schneider et al.	https://imagej.nih.gov/ij/
Inkscape	Inscape.org	N/A
NIS-Elements	Nikon	N/A
NZConnect software	Hamamatsu	Cat# U16414-01
Quantasoft	Biorad	N/A
Skyline software (version 23.1)	MacCoss Laboratory, University of Washington; Seattle, WA)	Open Source
SPSS Statistics Ver.22 software	IBM	https://www.ibm.com/products/spss-statistics?lot=5&mhsrc=ibmsearch_a&mhq=spss

**Other**		

Infinity UHPLC	Agilent Technology, Santa Clara, CA	1290
Protein G Dynabeads for immunoprecipitation	ThermoFisher Scientific, Waltham, MA	10009D
Triple quadrupole mass spectrometer	Agilent Technology, Santa Clara, CA	6495C
Zorbax Rapid Resolution High Definition (2.1 × 50 mm, 1.8 μm particle size) UHPLC column	Agilent Technology, Santa Clara, CA	959741–902

### Experimental model and subject participant details

#### Cell culture

HEK293T, NXS2, Fibroblasts from healthy and FRDA patients cells were maintained in Dulbecco’s Modified Eagle Medium (DMEM) GlutaMAX (Gibco), K562 cells were cultured in Roswell Park Memorial Institute (RPMI) 1640 medium (Gibco). Media were supplemented with 10% FBS and 1% penicillin/streptomycin (Gibco). Fibroblasts from healthy donors, GM23971 (Male, 33yo), GM23976 (Male, 22 yo) were obtained from the Coriell Cell Repository (NJ, USA) and H-normal (Male, 27yo) from Dr. Terry Roberts, Brunel University of London, with informed consent. Fibroblasts from FRDA patients FA1 (Female, 8yo), FA2 (Female, 11yo), FA3 (Female, 14yo) were provided by Dr. Aurélien Bayot (Université Paris, France); informed consent was obtained from patients and/or family members according to protocols approved by the Robert Debré Hospital ethical committee (Paris, France). Fibroblasts from FRDA patient GM04078 (Male, 30yo) were obtained from Coriell Cell Repository (NJ, USA). The mouse neuroblastoma NXS2 cell line was provided by Dr. Gianluca Sala, University G. D’Annunzio (Chieti, Italy).

#### Animals

Male and female YG8sR mice^30^ (typically with ∼200 GAA repeats in *FXN* intron 1) with the highest GAA repeats were bred through multiple generations to obtain mice with expanded GAA repeats (>700) as a result of intergenerational repeat instability/expansion^34^ (Figure S5). Up to 5 mice/cage were housed in environmentally enriched individually ventilated cages at 11-h dark versus 13-h light cycle, 20°C–23°C and 45–60% humidity. The mice were nourished with a diet of SDS RM3 expanded food pellets and standard drinking water. One week pre-transplantation and nine weeks thereafter, mice were temporarily given a wet diet in addition and antibiotic treated water. All procedures were carried out in accordance with the UK Home Office ‘Animals (Scientific Procedures) Act 1986’ and with approval from the Brunel University Animals Welfare and Ethical Review Board. For GAA repeat genotyping, genomic DNA was extracted from mouse ear clips by standard phenol/chloroform extraction and ethanol precipitation, and GAA PCR amplification.^57^^,^^58^ Primers are listed in the key resources table.

For experiments with congenic mice, male and female C57BL/6 (from Charles River) were housed in a 12-h day-night cycle with controlled temperature and humidity. The ventilated cages had sterile bedding and everyday supply of sterile food and water in the animal barrier facility at University College London. Experiments were conducted after approval by the University College London Animal Welfare and Ethical Review Body (project license PP1079892).

#### Human samples

HSPCs were isolated from the peripheral blood of three FRDA patients (patients characteristics, including sex and age, are indicated in Table S1), approved by the Bloomsbury Research Ethics Committee, ref. 13/LO/168. Normal control blood samples were collected complying with the Great Ormond Street Hospital Laboratory Medicine Quality Policy, Control Samples from Staff volunteers APOL 029; volunteers donors can opt out of providing biographic details; therefore the age and sex are not available for these samples. Human CD34^+^ HSPCs were also isolated from GCSF mobilised apheresis of a healthy donor (male, 28 yo; AllCells, Alameda, CA, USA) following standard immunomagenitc procedure using the human CD34 MicroBead Kit (Miltenyi Bioscience).

### Method details

#### Generation of frataxin fusion protein plasmid

The cDNA for human precursor frataxin or truncated frataxin fused to the secretion and APP or TAT sequences was synthesised by GeneArt (Invitrogen). The synthetic gene consisting of 5′-signal sequence-TAT or APP-FXN or truncated FXN-3′ DNA, with BamH1 restriction enzyme sites on each end was assembled from synthetic oligonucleotide or PCR products. The fragments were inserted into either the pLig GFP+ or the pCCL-PGK plasmids at BamH1 sites. The plasmid DNA was expanded and purified from transformed One Shot in Stbl3 competent E. coli bacteria (Thermo Fisher) using the NucleoBond Xtra Midi kit (Macherey Nagel), as per manufacturer’s instructions, and their sequence verified by DNA sequencing.

Secretion sequence: ATG GAC TTC CAG GTG CAG ATC TTC AGC TTC CTG CTG ATC TCC GCC AGC GTG ATC ATC AGC AGA GGC; APP peptide: ATG GCC TCT ATC TGG GTC GGA CAC AGA GGA; TAT peptide: - ATG TAT GGC CGC AAA AAA CGC CGC CAG CGC CGC CGC; frataxin precursor: - ATG TGG ACC CTT GGC AGA AGG GCC GTT GCT GGA CTG CTT GCC TCT CCA TCT CCT GCT CAA GCC CAG ACA CTG ACC AGA GTG CCT AGA CCT GCT GAA CTG GCC CCT CTG TGT GGC AGA AGA GGA CTG AGA ACC GAC ATC GAC GCC ACA TGC ACA CCT AGA AGG GCC AGC AGC AAT CAG AGA GGC CTG AAT CAG ATC TGG AAC GTG AAG AAA CAG AGC GTG TAC CTG ATG AAC CTG AGA AAG AGC GGC ACC CTG GGA CAC CCT GGA AGC CTG GAT GAG ACA ACC TAC GAG AGA CTG GCC GAG GAA ACC CTG GAT TCC CTG GCC GAG TTC TTC GAG GAC CTG GCC GAT AAG CCC TAC ACC TTC GAG GAT TAC GAC GTG TCC TTT GGC AGC GGC GTG CTG ACA GTG AAA CTC GGA GGC GAT CTG GGC ACC TAC GTG ATC AAC AAG CAG ACC CCT AAC AAA CAG ATC TGG CTG AGC AGC CCT AGC AGC GGC CCC AAG AGA TAT GAT TGG ACC GGC AAG AAC TGG GTG TAC AGC CAC GAT GGC GTG TCC CTG CAC GAA CTG CTG GCT GCC GAA CTG ACA AAG GCC CTG AAA ACA AAG CTG GAC CTG TCC AGC CTG GCC TAC TCT GGC AAA GAT GCC TGA AAT AAA; Truncated FXN: ATG TGG ACT CTC GGG CGC CGC GCA GAA AAG ATG CTT GAA ATA AAG.

#### Lentivirus production

HEK293T cells were co-transfected with empty or the *FXN-APP* insert containing pCCL plasmids along with pMDG2 (envelop vector) and pCMV-D8.74 (packaging vector) plasmids by polyethylenimine (Sigma Aldrich) and virus harvested from cell culture supernatants by ultracentrifugation and stored at −80°C. The viral titer was determined by infecting 2 × 10^5^ K562 cells with serial dilutions of the viral vector preparation and analysing vector copy number (VCN) by quantitative PCR of lentivector DNA (see VCN method below) in the cellular DNA isolates 3 days post transduction.

#### Verification of fusion protein production by cells transfected with frataxin fusion protein vectors

HEK293T cells were transfected with GFP+ or GFP- plasmids, with or without the frataxin fusion peptide DNA insert (TAT-FXN, APP-FXN, or EMP), or with the APP-truncated frataxin fusion peptide insert (APP-TRU), at 80–90% confluence, using Lipofectamine 3000 kit (ThermoFisher Scientific) or Polyethylenimine (PEI) transfection reagent and cells were incubated at 37°C, 5% CO_2_. The transfection efficiency was determined 48 h post transfection by flow cytometric quantification of GFP positive cells. Approximately, 1 × 10^6^ transfected cells were lysed in Laemmli buffer containing complete protease inhibitors (Roche) and acetone precipitated culture supernatants, were collected for western blot analysis and culture supernatants for FXN ELISA quantification, 72 h post transfection.

#### Verification of the incorporation of APP-FXN fusion protein in FRDA fibroblasts

Fibroblasts from healthy and FRDA patients were seeded at 1 × 10^6^/mL in 1mL of DMEM, 10% FBS and 1% penicillin/streptomycin in 6 well plates and incubated at 37°C, 5% CO_2_. After 24 h, the media was replaced with supernatant from the empty or fusion peptide positive plasmid transfected HEK293T cells, 72 h post transfection (see above) and further incubated for 2 and 4 h. Approximately, 1 × 10^6^ fibroblasts were lysed in Laemmli buffer containing complete protease inhibitors and assessed for their endogenous frataxin by western blot analysis.

#### Microscopy analyses and deep SIM imaging

Similarly, fibroblasts were analyzed by immunofluorescence microscopy for intracellular frataxin by staining 2 × 10^4^ supernatant treated cells seeded on coverslips with 100nM MitoTracker Red CMXRos (Invitrogen) for 30min at 37°C, fixing them in 4% paraformaldehyde then permeabilising for 10 min with PBS containing 0.2% Triton X-100. The samples were then incubated with a blocking solution of PBS 10% Goat Serum for 30 min hour at 37°C and stained with anti-frataxin antibody (18A5DB1, Abcam) for 16h at 4°C. The cells were incubated with appropriate fluorochrome tagged secondary antibodies (Abcam) at room temperature for an hour and then nuclear stained and mounted on slides with vectashield with DAPI (Vector Laboratories).

Samples were subsequently viewed using a spinning disk confocal NIKON Ti2-E inverted microscope with Teledyne Photometrics Kinetix camera through a 60x/1.42 DIC Plan Apo, oil immersion objective. The datasets were denoised with NIS Elements AR 5.42.06 (NIKON). Three-dimensional datasets were converted to a maximum projection, exported as PNG files and imported into Inkscape software (version 1.4) for final presentation. For deepSIM imaging, a NIKON Ti2-E inverted microscope was used with Teledyne Photometrics Kinetix camera and a Nikon 60x/1.42 DIC Plan Apo, oil immersion objective. 3D stacks were acquired and reconstructed with the NIS Elements AR 5.42.06 (NIKON). Single plane images were exported as PNG files and imported into Inkscape software (version 1.4) for final presentation.

#### H_2_O_2_ survival assays

To test for the effects of treatment with frataxin fusion peptide on fibroblast viability following H_2_O_2_ induced oxidative stress, the MTS assay was utilised. Briefly, FRDA and normal fibroblast cells were seeded in a 96-well plate containing 100μL of DMEM, 10% FBS and 1% penicillin/streptomycin at 20,000 cells per well overnight at 37°C, 5% CO_2_. The media was changed and replaced with 100μL of supernatant from the PEI only, the empty or fusion peptides positive plasmid transfected HEK293T cells (see above). After 1 h, 0, 50, 200, 400, 500and 600 μM (as shown in each figure) of hydrogen superoxide (H_2_O_2_) (ACROS Organics) was added to cells in triplicate wells over 6 h to induce oxidative stress. Cell viability was subsequently assessed by MTS assay using the CellTiter 96 AQueous Non-Radioactive Cell Proliferation (Promega), in accordance with the manufacturer’s protocol, with colorimetric readings obtained using ELx808 microplate reader (Biotek) or SpectraMax i3x (Molecular Devices).

In order to analyze the effects of frataxin fusion peptides on aconitase activity levels in FRDA fibroblasts, 1 × 10^6^ cells from FA and healthy individuals were incubated for 1 h with supernatant collected from HEK293T cells that had been transfected with frataxin fusions peptide constructs or empty vector control. Fibroblast were lysed and aconitase measured using the Aconitase Assay kit (Abcam) as described below.

#### Validation of secretion and penetration activity of the APP-FXN fusion protein

HEK293T cells (5 × 10^5^) were seeded and transfected the following day with plasmids encoding human FXN^wt^ or human APP-FXN, together with a GFP plasmid to monitor transfection efficiency. Transfection efficiency was assessed by flow cytometry. Forty-eight hours post-transfection, part of the supernatant was collected and reserved for frataxin measurement, and the remaining supernatant was transferred to 1 × 10^6^ mouse bone marrow cells or NXS2 mouse neuroblastoma cells. After 2 h of incubation, frataxin levels were measured in both the reserved supernatant and the recipient cells using the Human Frataxin SimpleStep ELISA Kit (ab176112) according to the manufacturer’s instructions.

For analysis, secreted frataxin levels in the supernatant were first normalized to the number of GFP-positive transfected cells and then expressed relative to GFP-only transfected controls. For the recipient cells, frataxin levels were presented as a ratio relative to GFP control for both mouse bone marrow and NXS2 cells.

#### Transduction of human CD34^+^ HSPCs

Human CD34^+^ HSPCs from GCSF mobilised apheresis of healthy donors were seeded at 1 × 10^6^/mL in StemSpan medium (STEMCELL Technologies), 1% penicillin/streptomycin (Thermo fisher) with full cytokine cocktail; 100 ng/mL hFlt3-Ligand (hFlt3L) 100 ng/mL hStem Cell Factor (hSCF), 20 ng/mL hThrombopoietin (hTPO) (all Peprotech). Following cytokine pre-stimulation for 24 h, cells were seeded in StemSpan medium, 1% penicillin/streptomycin, hSCF 50ng/ml, hFlt3L 50ng/ml, hTPO 10 ng/mL, hInterleukin-3 (hIL3) 20ng/ml (all from Peprotech) at a concentration of 2 × 10^6^ cells/ml in 24-well plates and fusion peptide plasmid construct positive virus (MOI = 20) and 4μg/ml protamine sulfate (Thermo Fisher Scientific) added to the cells. Twenty-four hours post transduction, cells were cultured in semisolid media and red/white colonies were scored after for 14 days.

FRDA CD34^+^ cells were grown for one week in StemSpan medium with hSCF 100ng/ml, hFlt3L 100ng/ml, hTPO 20 ng/mL, 20 ng/mL hIL-6, 60 ng/mL hIL-3 (all from Peprotech) and in the presence of of 1μM StemRegenin and 50 nM UM171 (both from STEMCELL technologies) to maintain stemness. Cells were transduced with the lentiviral vector expressing the modified frataxin protein for 24 h (MOI = 20), in the presence of 1mg/ml of LentiBOOST (Revvity Gene Delivery GmbH) and 4μg/ml protamine sulfate. Patient cells, alongside with healthy donors CD34^+^, were cultured in macrophage differentiation medium-Iscove’s Modified Dulbecco’s medium (IMDM) supplemented with hSCF (20 ng/mL), hFLT3-ligand (30 ng/mL), hIL-3 (30 ng/mL), human macrophage colony stimulating factor, hM-CSF (30 ng/mL) and FBS (20%) at 37°C/5% CO_2_ for 10 days and in RPMI (10% FBS) with hM-CSF (50ng/ml) for further 2 weeks. Secreted frataxin in cell supernatants was quantified by ELISA assay.

#### Isolation and transduction of mouse Lin-HSPCs

Femurs and tibias were dissected from male donor mice (either FRDA or C57BL/6) and bone marrow cells obtained by flushing with RPMI (Gibco) media. Cells were pelleted and enriched for Ter119^-^, Gr1, Mac1^-^, B220^-^, CD4^−^, CD8^−^, IL7R^−^ and Sca^+^, cKit^+^ cells by magnetic cell sorting using mouse Lineage Cell Depletion Kit (Miltenyi Biotech) according to the manufacturer’s protocol. Flow cytometric analysis for cKit expressing cell levels revealed the percentage of cKit^+^ cells increased from approximately 9%–56% of total population post cell sorting (data not shown). Isolated HSPCs were seeded at 1 × 10^6^/mL in 1x culture StemSpan media (Stem Cell Technologies), 1% penicillin/streptomycin (Thermo fisher) with full cytokine cocktail; mSCF 100ng/ml, mFlt3L 100ng/ml, mTPO 20 ng/mL. Following cytokine pre-stimulation for 4 h, cells were seeded in Stem Span medium, 1% penicillin/streptomycin, mSCF 50ng/ml, mFlt3L 50ng/ml, mTPO 10 ng/mL, mIL3 20ng/ml at a concentration of 2 × 10^6^ cells/ml in 24-well plates and fusion peptide plasmid construct positive plasmid containing virus (MOI = 6 or 12.5) added to infect the cells for at least 14 h. HSPCs were extensively washed and reconstituted in PBS at 0.5 × 10^6^ cells/100μL for transplantation. A portion (0.5 × 10^6^) of the cells were plated into 48 well plates in Stem Span medium, 1% penicillin/streptomycin, mSCF 50ng/ml, mFlt3L 50ng/ml, mTPO 10 ng/mL, hIL3 20ng/ml and cultured for 5 days and 0.1 × 10^6^ cells were used for DNA purification using the Monarch Genomic DNA Purification Kit (New England Biolabs) according to manufacturer’s instruction to test vector copy numbers as described below. Another 0.5 × 10^6^ of the cells were lysed to assess their frataxin content by western blot.

#### Assessement of transduction efficiency and engraftment levels

Average vector copy number/cell (VCN) was analyzed by droplet digital PCR. DNA samples were diluted in water to 10ng/μL. Reactions were performed in a final volume of 22 μL, containing 1x ddPCR Supermix for probes (BioRad, Hercules, CA), 0.8μL of each primer (10μM) and 0.4μL of each probe (10μM), 2μL of DNA per sample and 5μL of nuclease free water. 20,000 droplets were generated using the automated droplet generator (BioRad) before amplification with C1000 Touch Thermal Cycler; 96–Deep Well Reaction Module (BioRad). Positives and negatives droplets were quantified using the QX200 Droplet reader (BioRad) and analyzed by Poisson statistics using QuantaSoft Software (BioRad). Concentration was provided by Quantasoft software as copies of the relevant gene per μL (copies/μL).VCN was calculated as the ratio between the target gene concentration (h*FRATAXIN*) and the reference gene concentration (m*Titin*) x2: hFXN copies/mTitin copies ∗2 or Albumin for human samples. Primers and probes are listed in Table S3.

Levels of engraftment (male cells into female hosts) were determined by looking at the Y chromosome copies. *TSPY* male specific DNA assessment by real-time PCR was performed using pre-designed TSPY primers/probe set (#4426961, Thermo Fisher) and normalised with *b-actin* DNA using pre-designed primer/probe set (#4331182 Thermo Fisher) using TaqMan Fast Advanced Master Mix (#4331182 Thermo Fisher) according to manufacturer’s protocol. Fold difference for each female mouse DNA sample was determined by comparison with male DNA as a reference via the delta-Ct method^59^ and subsequently converted as a percentage of the male DNA TSPY gene level (at 100%).

#### YG8sR mice transplantation and behavioral studies

YG8sR female mice were treated with approximately 0.5 × 10^6^ male syngeneic HSPCs, transduced with frataxin fusion peptide lentiviral vector (LV) or WT HSPCs via intravenous tail vein injection, from eight weeks of age following total body lethal irradiation (2 × 5 grays with 3–4 h interval). Two to three days prior irradiation, all mice were assessed for their baseline beam walk, activity and rotarod performance at eight weeks of age and then randomised into their treatment groups (WT untreated [B6] *n* = 5; YG8sR untreated/untransplanted [UTX] *n* = 9; YG8sR-WT HSPC transplanted [WTX] *n* = 6; LV-FXN (i.e., infected with ∼12.5 MOI lentivirus) YG8sR-fusion peptide+ LV HSPC transplanted [LV-FXN high] *n* = 9, low (i.e., infected with ∼6 MOI lentivirus) YG8sR-fusion peptide+ LV HSPC transplanted [LV-FXN low] *n* = 7). Body weights were monitored weekly from 7 weeks of age pre-transplantation and twice weekly post transplantation for four weeks and then weekly thereafter to detect potential adverse effects of treatment. Mice displaying reduction in body weight by 20%, with other measures of appearance and behavior indicating ill-health were euthanised, a total of six mice were euthanised with postmortem analysis indicating lung infection to be the likely cause of illness. Normal blood cell development and reconstitution post transplantation and no signs of T cell, B cell or monocyte/macrophage/dendritic cell tumorigenesis were observed after flow cytometric analysis of CD3, CD19 or CD11b positive splenic and blood cells respectively from these mice. Efficacy of treatment was investigated at eight (pre-treatment) and sixteen weeks of age, and then every four weeks thereafter up to forty-eight weeks of age by measuring beam walk and rotarod performance, and exploratory activity in the open field test as previously described.^30^^,^^60^ Different tests were undertaken on separate days. Beam walk test was performed three times, after an initial trial run, by each mouse over a day, with a rest period of at least ten mins between each run. The time taken for the mice to traverse from one end of the 18mm thick beam to the other end, 90cm away, was taken and mean time to traverse were calculated for each treatment group. For each rotarod trial, mice were allowed to acclimatise to the rotating drum for 10 sec before it began to accelerate from 4 to 40 rpm for a maximum of 300 sec. Mice were tested for four trials per day, with the first trial used as a practice run. Mice were rested for a minimum of 10 min between each trial. The mean latency to fall times for each mouse group at specified time points were calculated as described previously.^30^^,^^60^ Exploratory/locomotor activity in the open field test was measured by placing mice individually in a beam-breaker activity monitor box (MEDOFA-510 activity chamber; Med Associates) for 4 mins to assess exploratory behavior in a novel environment. Mice were assessed for four trials per day, with the first trial used as a practice run. Mice were rested for a minimum of 30 min between each trial. The average total distance traveled (cm) and mean velocity (cm/s) of each mouse was recorded and means for each mouse treatment group were calculated.

#### Blood/plasma, white blood cell and tissue sample collection

Blood was taken via tail vein puncture into EDTA tubes. Blood samples were spun at 1000 × *g* for two mins and the upper plasma layer removed for human FXN ELISA quantification. The pelleted blood cells were treated with mouse red blood cell lysis solution (R&D Systems) in order to remove red blood cells and DNA was extracted from the remaining leukocytes samples using the Monarch Genomic DNA Purification Kit (New England Biolabs) according to the manufacturer’s protocol for VCN and *TSPY* PCR analysis. Following euthanasia by pentobarbitol injection, mice were perfused with PBS and leg muscle (soleus), heart and cortex tissues were dissected and snap frozen for subsequent storage at −80°C, and cerebellum fixed in 4% paraformaldehyde (PFA) overnight then replaced with PBS sodium azide and stored at 4°C.

#### Transplantation experiments with congenic mice

Bone marrow was harvested from femur and tibias of B6.SJL-Ly5.1 mice (expressing CD45.1 allele) and lineage negative HSPCs were isolated via magnetic column separation (Miltenyi). Cells were then transduced with the pCCL-APP-frataxin WPRE lentiviral vector (LV-FXN) with a MOI of 20 for 24 h in StemSpan medium with mSCF 100ng/ml, mFlt3L 100ng/ml, mTPO 20 ng/mL. The day after cells were resuspended in 200μL of PBS and injected into the tail vein of 6–7 weeks of age C57BL/6-Ly5.2 congenic mice (expressing the CD45.2 allele) that were given a lethal split dose of irradiation, 5 Gy followed by 4 Gy the day after. The mice were divided into 2 groups Mock untransduced (*n* = 3) and vector transduced (*n* = 4). Experiments were approved by the University College London Animal Welfare and Ethical Review Body (Project license 70/8241).

#### Flow cytometry

Transfected HEK293T cells were analyzed for GFP expression via flow cytometry using the Novocyte flow cytometer (ACEA). Briefly, 0.5 × 10^6^ cultured cells were harvested 48 h post transfection and fixed in 2% paraformaldehyde (PFA). For the viral infected CD34^+^ cells differentiated over 22 days, 0.5 × 10^6^ cells were stained with anti-human CD11b-APC antibody (M1/70, Thermofisher) for 1 h at 4°C and fixed in 2% PFA. For the magnetic cell sorting (MACS) enriched bone marrow derived mouse HSPCs purity analysis, 0.5×10^6^ cells were stained with anti-mouse cKit (CD117)-FITC antibody (Biolegend) for 30 min on ice and fixed in 2% PFA. Blood, bone marrow and splenic cells were stained with antibodies for 30 min on ice and fixed in 2% PFA and analyzed for T cell (anti-mouse CD3-pacific blue antibody, Biolegend), B cell (anti-mouse CD19-FITC antibody, Biolegend) and monocyte/macrophage and dendritic cell (anti-mouse CD11b-APC antibody, Biolegend) contents in order to assess leukocyte development of transplanted HSPC and detect signs of tumorigenesis in these cells. Fixed/stained cells were ran on the flow cytometer acquiring 20,000 cells (gated according to forward and side scatters with doublets excluded according to FSC A/FSC-H dot plots). Corresponding untransfected or unstained cell sample controls were acquired to establish the gating areas for positively staining cells. For the experiments with congenic mice, cells were stained with the following antibodies: anti-mouse CD45.2-FITC, anti-mouse CD45.1-PE, CD45R (B220)-APC (all from Miltenyi) anti mouse CD11b APC-Cyanine7(Biolegend), anti mouse CD3-SBV440(Biorad). Samples were acquired using the LSRII flow cytometer (BD) and analyzed using FlowJo10 analyser (BD).

#### Western blotting

Tissue (Tris HCL lysed) or cell (Laemmli lysed) protein lysates and culture supernatants were sonicated at 4°C using a vibracell sonicator (10 × 1 s 20 kHz pulses) and denatured for 10 min at 95°C, loaded onto 15% SDS polyacrylamide gels, transferred onto nitrocellulose membranes and subjected to western blotting. Membranes were blocked in PBS 5% milk containing Tween 20 (BBT) overnight at 4°C. Primary antibodies against frataxin (ab110328, AB113691Abcam), actin (C-2, Santa Cruz) or GAPDH (1E6D9, Proteintech or sc-166574, Santa Cruz) were incubated overnight at 4°C in BBT. Blots were washed three times for 5 min in PBS Tween 20 (PBST) and incubated for 1 hr at RT with the appropriate secondary antibodies conjugated with horseradish peroxidase (HRP). Blots were washed three times in PBST, and the target protein visualised on Amersham Hyperfilm ECL films (GE Healthcare) using enhanced chemiluminescence (ECL) reagents (BioRad) according to the manufacturer’s instructions and a film processing unit (Xograph). Frataxin protein bands were quantified using Image Studio (LI-COR), target protein bands were normalised with their corresponding actin or GAPDH protein bands or with total protein via Ponceau S staining.

#### ELISA assay

Culture supernatant, protein lysate, tissue homogenates or plasma human frataxin contents were quantified using the frataxin ELISA kit (Abcam) according to the manufacturer’s protocol. Colorimetric measurements of ELISA plates were performed using the ELx808 microplate reader.

#### Aconitase assay

Cells were washed with cold PBS and suspended in 100μL of cold Assay Buffer (Abcam). This was followed by centrifugation at 800*g* for 10 min at 4°C. Activated aconitase in the lysates were quantified using the Aconitase Assay kit (Abcam) according to the manufacturer’s instructions.

Frozen 20g mouse heart, muscle and cerebellum tissues were homogenised in cold Aconitase Assay kit Assay Buffer (Abcam), centrifuged at 800g for 10min at 4C. Activated aconitase in the lysates were quantified using the Aconitase Assay kit according to the manufacturer’s instructions and normalised to citrate synthase activity measured via 5,5′-dithiobis-(2-nitrobenzoic acid) (DTNB) reduction analysis. Briefly, tissue extracts were added to Tris–HCl 100 mM pH 8.1 with 0.4 mg/mL DTNB and 10 mg/mL Acetyl-CoA. Colorimetric analysis of aconitase and citrate synthase plates at 450nm and 412nm respectively were performed with the ELx808 microplate reader.

#### Histology

For histological analysis, mice were anesthetized and perfused with PBS. Brains were dissected and submerged in 4% PFA in PBS for 24 h at 4°C then in PBS sodium azide at 4°C until processing. Tissues were embedded in paraffin for sectioning on a rotary microtome and mounted on glass slides. Slides were deparaffinized, rehydrated, Harris Modified Hematoxylin (H) stained and then blocked with 10% normal goat serum diluted in 0.1% Triton X- PBS. For beta III tubulin staining, sections were incubated at 4°C overnight with primary antibody to beta III tubulin (ab78078, Abcam) followed by 1 h at room temp biotinylated anti-mouse antibody (DAKO). For FXN staining, sections were incubated at room temp for 60 min with primary antibody to FXN (ab219414, Abcam) followed by 1 h at room temp biotinylated anti-rabbit antibody (ab207995, Abcam). Immunohistochemistry staining was performed using the Ventana Discovery XT instrument, using the Ventana DAB Map detection Kit (760–124). For pre-treatment, Roche Cell Conditioning Solution CC2 (950–123) for beta III tubulin analysis and Roche Cell Conditioning Solution CC1 (950–500) for FXN staining were used. Slides were scanned using the Hamamatsu Nanozoomer S360 Digital slide scanner and scanned at x40 magnification and viewed/analyzed with NZConnect software (Hamamatsu). All histological work was performed at the IQ-Path Lab, UCL Institute of Neurology, UK.

#### hFXN-M immunoprecipitation (IP)

IP was performed following a previously described protocol with minor modifications.^61^ Briefly, a portion of tissue homogenate (typically 500 μL) was mixed with 500 μL of ice-cold RIPA lysis buffer (supplemented with 1 × complete protease cocktail,1 mM DTT). Mature SILAC-hFXN (50 ng) containing [^13^C_6_]-Leucine was spiked into each sample to serve as the internal standard. Each sample was transferred to a 2 mL LoBind Eppendorf tube containing 100 μL protein G magnetic beads (0.5 mg) cross-linked to an anti-FXN recombinant rabbit monoclonal antibody (EPR21840, ab219414, Abcam, Waltham, MA) with dimethylpimelimidate and then incubated on a rotator at 4°C overnight. Beads were washed and then eluted with 300 mM acetic acid in 10% acetonitrile. Dried samples were dissolved in 50 μL 25 mM aqueous NH_4_HCO_3_ solution containing 500 ng trypsin protease and digestion was performed at 37°C overnight prior to ultra-high performance liquid chromatography-multiple reaction monitoring/mass spectrometry (UHPLC-MRM/MS) analysis. Calibration standards were prepared in 5% bovine serum albumin, and analysis of the calibration standards was performed alongside the samples following the same protocol. The linear standard curve obtained for the specific hFXN-M N-terminal tryptic peptide (S^81^GTLGHPGSLDETTYER^97^) was used to calculate hFXN-M levels over a 1–50 ng range.

#### UHPLC-MRM/MS and data analysis

Samples were analyzed using an Agilent 1290 Infinity II UHPLC system interfaced with a 6495C triple quadrupole mass spectrometer (Agilent Technologies Inc., Santa Clara, CA) as described previously^62^ Injections of 2 μL were made, the injector was held at 4°C, and the needle flushed for 5-s with 30% methanol. Solvent A was water containing 0.1% formic acid, and solvent B was acetonitrile containing 0.1% formic acid. The Zorbax Rapid Resolution High Definition (2.1 × 50 mm, 1.8 μm particle size) UHPLC column was maintained at 35°C with a flow rate of 0.4 mL/min. Analytes were eluted with a linear gradient from 95% A at 0-min to 5% A over 8.0-min. Ionization was conducted using the Agilent Jet Stream ESI source. Protein quantification was performed using Skyline software (version 23.1; MacCoss Laboratory, University of Washington; Seattle, WA).^63^ The peak area ratio of the three most intense MRM/MS transactions for unlabeled/light (L) peptide to labeled/heavy (H) peptide was calculated by Skyline software and used for absolute quantification.

### Quantification and statistical analysis

Differences between specified groups were detected using Student’s *t* test, linear regression, or analysis of variance (ANOVA) test with *post hoc*, Scheffé's or Bonferroni’s correction for multiple comparison test where appropriate (PRISM and IBM SPSS Statistics Ver.22 software). All data were screened for statistical outliers using Grubbs test (GraphPad Software) and outlier values were excluded from the analysis. *p* values of <0.05 were considered significant.

## Supplemental information

Document S1. Figures S1–S5 and Tables S1–S3

Document S2. Article plus supplemental information
