# Supplementary material for: Therapeutic activity of a hematopoietic stem cell-delivered cell-penetrating frataxin in Friedreich’s ataxia models
Source: Cell Rep Med. 2026 May 13;7(6):102803. doi: 10.1016/j.xcrm.2026.102803 (PMC13293935; doi:10.1016/j.xcrm.2026.102803)
Supplement: Document S1. Figures S1–S5 and Tables S1–S3 [file mmc1.pdf]

Cell Reports Medicine, Volume *7*

Supplemental information

Therapeutic activity of a hematopoietic stem cell-delivered cell-penetrating frataxin in Friedreich's ataxia models

Jeffrey Pido-Lopez, Shefta E Moula, Enas Shaban, Konstantinos Stamatiou, Bethan J. Critchley, Thomas E. Whittaker, Stina Svensson, Sara Anjomani-Virmouni, Ester Kalef-Ezra, Lucinda Carr, Jane Hassel, Adrian J. Thrasher, Manju A. Kurian, Ian A. Blair, Teerapat Rojsajjakul, Giorgia Santilli, and Arturo Sala

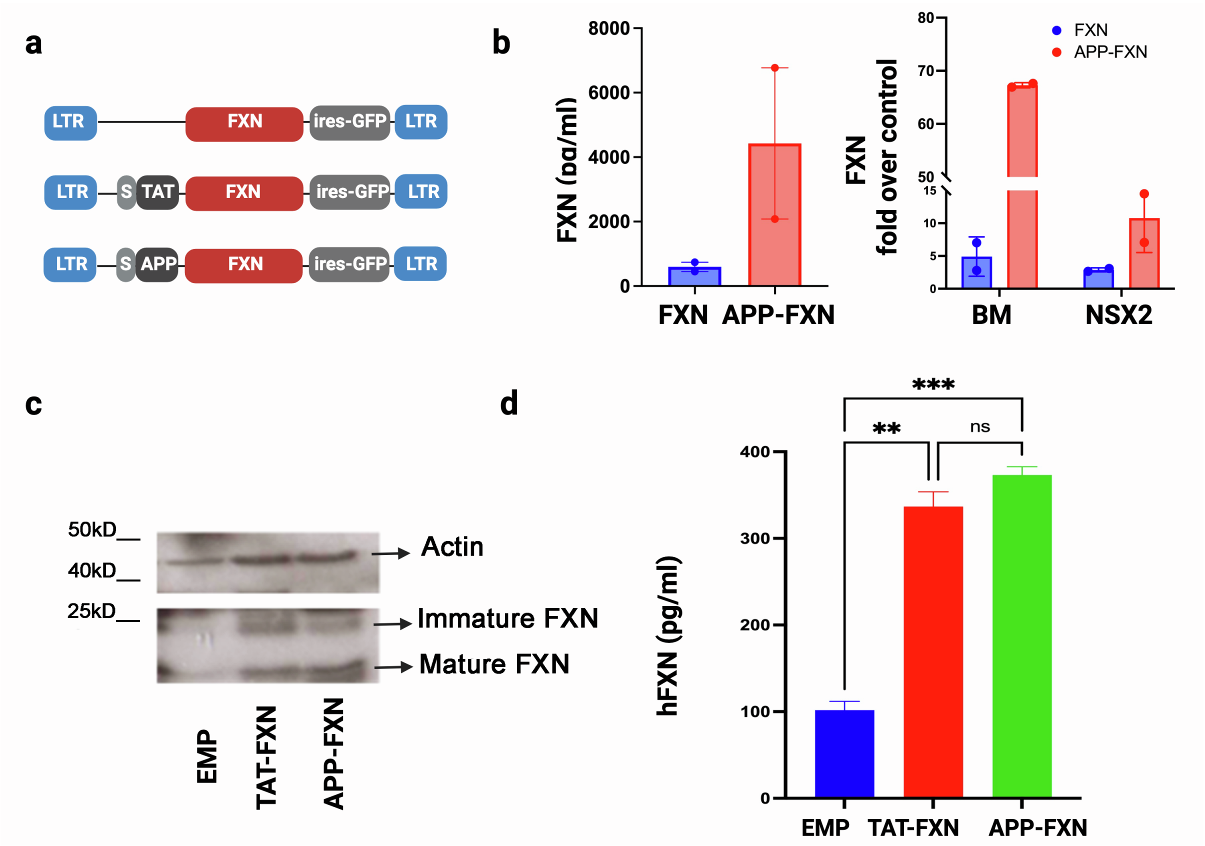

Supplementary Figure 1. Characterisation of secretion and cell penetration of the Frataxin fusion peptides. a) schematic representation of the structure of the pLIG lentiviral constructs encoding wild type Frataxin or secreted Frataxin linked to the TAT or APP tissue penetrating peptides; **b**) HEK293T cells were transfected with GFP (control), FXN^{wt}, or APP-FXN. Secreted Frataxin in the supernatant was measured by ELISA 48 h post-transfection (left panel). Supernatants from FXN- or APP-FXN–transfected HEK293T cells were transferred to mouse bone marrow or NXS2 cells for 2 h. Frataxin levels in recipient cells were measured by ELISA and expressed as a ratio relative to GFP control. Data represent the mean of two biological replicates; error bars indicate SEM (right panel). (**c**) western blotting for FXN protein in lysates of cells transfected with either empty (EMP) plasmid, TAT-FXN or APP-FXN plasmid constructs, representative blot of two separate experiments. (**d**) HEK293 cells transfected with plasmid containing the FXN-TAT or FXN-APP gene constructs produced and secreted cell penetrating fusion proteins as revealed by human FXN levels in supernatants from transfected cell cultures as measured by ELISA; mean values ±SEM are shown; *N*=3/group, ** p <0.01, ***p <0.001 by multiple comparison ANOVA test with Bonferroni's post hoc analysis.

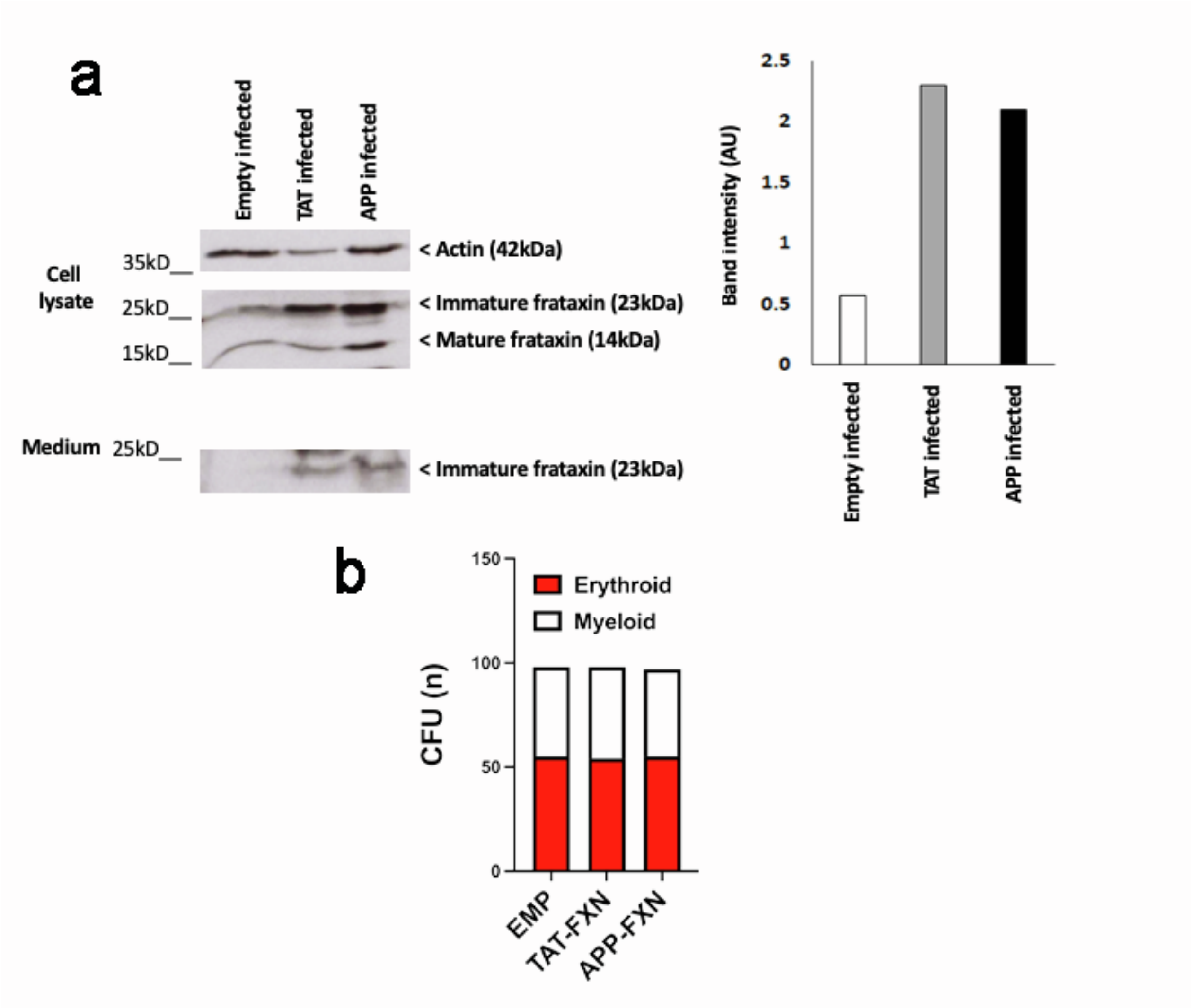

Supplementary figure 2. Transduction of CD34+ HSPCs with pLIG lentivirus containing FXN-TAT or FXN-APP fusion proteins. (**a**) Lysates of CD34+ HSPCs infected with the empty vector (EMP), or FXN-TAT, or FXN-APP plasmid positive lentivirus were assessed for their frataxin contents by western blotting. Bar graph shows quantification of mature FXN bands' intensity normalised with actin. (**b**) colony forming units (CFU) after 14 days of semisolid cultures of CD34+ HSPCs transduced with the indicated viruses in myeloid differentiation conditions.

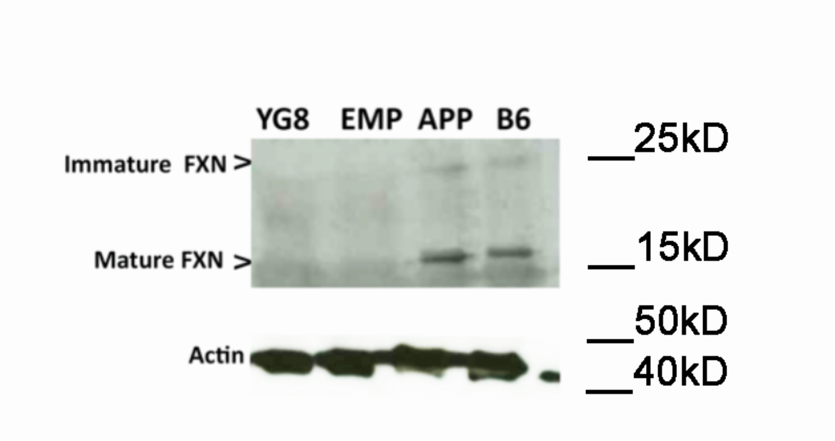

Supplementary figure 3. Expression of frataxin after infection of YG8sR HSPCs with the pCCL PKG FXN vector (APP) compared to untransplanted (YG8) or empty vector (EMP); HSPCs from a normal BL6 mouse (B6) served as a positive control for frataxin expression.

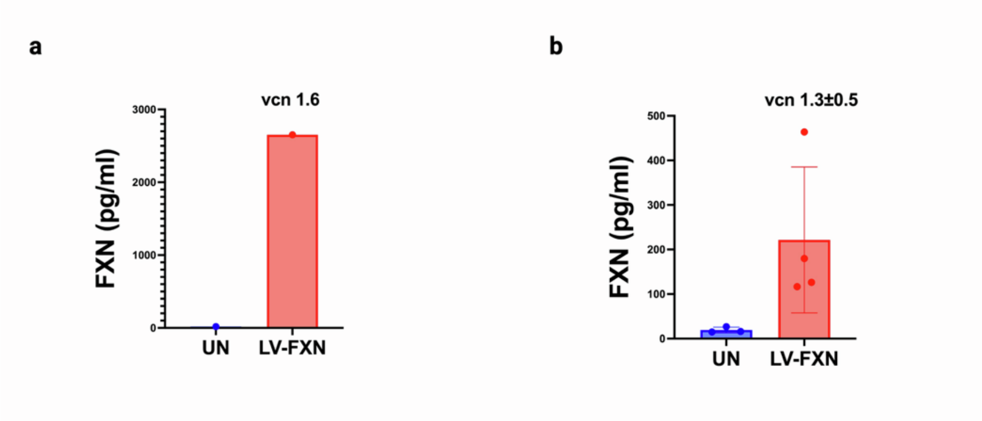

Supplementary Figure 4. Plasma levels of Frataxin in transplanted congenic mice. HSPCs (Lineage negative) isolated from LY5.1 donors were transduced with LV-FXN at MOI 20 and transplanted into LY5.2 recipients. Levels of Frataxin in the supernatant of cells 5 days post transduction and vector copy numbers (vcn) are shown in (**a**). Average levels of Frataxin in plasma of transplanted animals at termination (3 months post transplantation) and average±SD vector copy numbers are shown in (**b**).

a

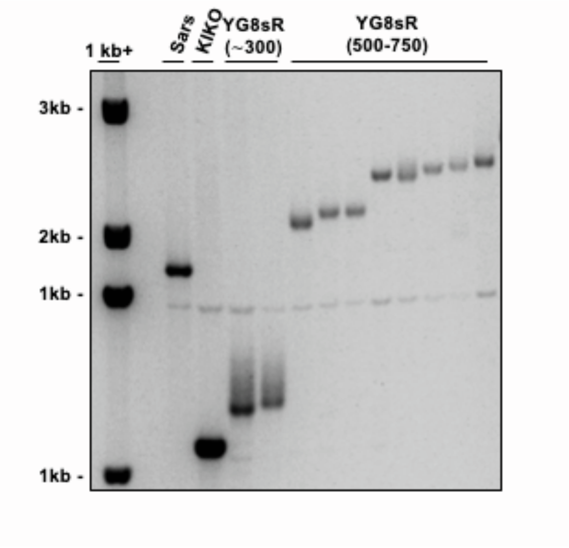

b

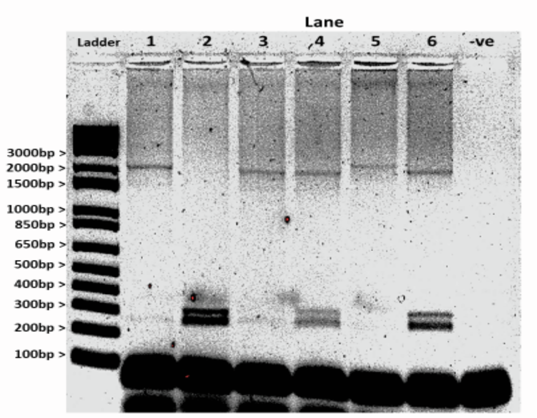

Supplementary figure 5. Characterization of YG8sR mice. (**a**) GAA repeat analysis, representative example of GelRed stained agarose gel (1.5%) showing the PCR analysis of GAA repeat in ear clip tissues of Sarsero (Sars) (lane 3), KIKO (lane 4), YG8sR$(\mathrm{GAA}){\sim}300$ (lanes 5-6), YG8sR$(\mathrm{GAA}){\sim}500$ (lanes 7-9), YG8sR$(\mathrm{GAA}) > 700$ (lanes 10-14). 1 kb+ (1 kb plus ladder) and 100 bp (100 bp DNA ladder) were used as molecular markers; (**b**) Mice genotyping, GAA PCR of genomic DNA shows mice with bands at 300bp for hemizygous Y47R mice (in lane 2), >2000bp for hemizygous YG8sR mice (in lanes 1, 3, 5) and both 300 and >2000bp for heterozygous Y47R/YG8sR mice (in lanes 4, 6) generated from breeding hemizygous Y47R mice with hemizygous YG8sR mice.

Supplementary Table 1. Patients characteristics

Patient	*FXN* GAA repeats	Age at presentation and gender	Current age	Presenting clinical features	Clinical course	Investigations
1	Not reported	8y<br>Female	16y	Longstanding history of clumsiness presenting at 8 years with areflexia.	Still walking with support at 16y<br>Using powered mobility outside<br>Brief period of functional neurological illness with abnormal movements and non-epileptic seizures<br>Scoliosis surgery at 12 years<br>-orally fed<br>Pes cavus<br>Dysarthria present<br>No significant visual difficulties<br>Severe fatigue	MRI brain scan – unremarkable<br>EMG - severe mainly sensory generalised axonal neuropathy<br>Echocardiogram – mild asymptomatic hypertrophic cardiomyopathy<br>Video fluoroscopy - aspiration risk
2<br>Sibling of Pt 3	Not reported	5y<br>Female	8y	Presymptomatic diagnosis in light of sibling's presentation<br>Ataxia evident from 5y	Still walking independently<br>Areflexic<br>Mild scoliosis<br>Not dysarthric<br>No pes cavus<br>No significant visual issues	Echocardiogram - hypertrophic cardiomyopathy, on Atenolol
3<br>Sibling of Pt 2	Not reported	6yrs<br>Male	10yrs	Presented at 6yrs with ataxia and areflexia	At 10yrs still walking with support indoors, wheelchair outside<br>Mild scoliosis<br>Pes cavus<br>Dysarthria present<br>No visual difficulties<br>Severe fatigue	MRI brain scan - normal<br>EMG - absent sensory nerve action potential<br>Echocardiogram - hypertrophic cardiomyopathy, on Atenolol

Supplementary Table 2. Blood counts of single mice transplanted with HSPCs modified with the different lentivirus. F= High titer Frataxin; f= low titer Frataxin; W= wild type HSPC; U=unttransplanted.

MOUSE Blood @35 wks old	% B cells (CD19+)	% Myeloid (CD11b+)	% T cells (CD3+)	WBC count/ul
U570	51	20	32	5600
U639	37	24	36	3200
W526	63	21	12	6100
W527	16	58	12	5900
f404	33	43	14	2700
f455	62	20	11	6200
f457	63	18	16	3400
f487	29	56	8	2300
f534	53	16	15	1900
f573	39	30	22	6500
f587	21	12	8	1900
F354	51	20	32	5600
F381	37	24	36	3200
F388	30	23	38	3500
F496	49	17	23	3500
F505	57	16	19	4500
F557	51	19	13	3300
F641	51	15	31	3500
F645	28	14	24	1500
**Mouse Bone Marrow@48wks**	**% B cells (CD19+)**	**% Myeloid (CD11b+)**	**% T cells (CD3+)**	
U445	4	53	9	
U640	14	47	6	
W510	7	28	21	
W513	7	59	8	
W526	8	32	14	
f457	3	64	6	
f534	7	52	8	
F496	8	64	10	
F505	4	60	10	
F557	4	24	21	
F587	2	67	5	
F641	15	52	12	
F645	8	55	13	
**Mouse Spleen @48 wks**	**% B cells (CD19+)**	**% Myeloid (CD11b+)**	**% T cells (CD3+)**	
U445	7	18	26	
U640	31	5	16	
W510	6	12	16	
W513	7	8	29	
W526	12	6	30	
f457	3	10	14	
f534	22	7	30	
F496	10	12	22	
F505	5	6	10	
F557	5	20	21	
F587	6	7	26	

Supplementary Table 3. Primers and probes used for the assessment of viral transduction efficiency and engraftment levels, (Related to STAR methods).

Name	Sequence
ddPCR_Albumin_Forward	GCTGTCATCTCTTGTGGGCTG
ddPCR_Albumin_Reverse	ACTCATGGGAGCTGCTGGTTC
ddPCR_Albumin_Probe	**HEX**/CCTGTCATG/**ZEN**/CCCACACAAATCTCTCC/**BHQ**
ddPCR_Titin_Forward	ACCGAGAGAGGTGGTATTGA
ddPCR_Titin_Reverse	AGGATGCCTCCTGCTTAGA
ddPCR_Titin_Probe	**HEX**/AGCGTCTCG/**ZEN**/TCTCAGTCAGTCCAA/**BHQ**
ddPCR_FTX_Forward	GACCTGCTGAACTGGCCC
ddPCR_FTX_Reverse	GCCTCTCTGATTGCTGCTGG
ddPCR_FTX_Probe	**FAM**/CTGAGAACC/**ZEN**/GACATCGACGCCACATGCACACC/**BHQ**
